# Effect of Silver Nanoparticles on the In Vitro Regeneration, Biochemical, Genetic, and Phenotype Variation in Adventitious Shoots Produced from Leaf Explants in Chrysanthemum

**DOI:** 10.3390/ijms23137406

**Published:** 2022-07-03

**Authors:** Alicja Tymoszuk, Dariusz Kulus

**Affiliations:** Laboratory of Ornamental Plants and Vegetable Crops, Faculty of Agriculture and Biotechnology, Bydgoszcz University of Science and Technology, Bernardyńska 6, 85-029 Bydgoszcz, Poland; dariusz.kulus@pbs.edu.pl

**Keywords:** *Chrysanthemum* × *grandiflorum* (Ramat.) Kitam., induced mutagenesis, molecular markers, nanotechnology, oxidative stress, phenotype alternation

## Abstract

Novel and unique properties of nanomaterials, which are not apparent in larger-size forms of the same material, encourage the undertaking of studies exploring the multifaced effects of nanomaterials on plants. The results of such studies are not only scientifically relevant but, additionally, can be implemented to plant production and/or breeding. This study aimed to verify the applicability of silver nanoparticles (AgNPs) as a mutagen in chrysanthemum breeding. *Chrysanthemum* × *grandiflorum* (Ramat.) Kitam. ‘Lilac Wonder’ and ‘Richmond’ leaf explants were cultured on the modified MS medium supplemented with 0.6 mg·L^−1^ 6-benzylaminopurine (BAP) and 2 mg·L^−1^ indole-3-acetic acid (IAA) and treated with AgNPs (spherical; 20 nm in diameter size; 0, 50, and 100 mg·L^−1^). AgNPs strongly suppressed the capability of leaf explants to form adventitious shoots and the efficiency of shoot regeneration. The content of primary and secondary metabolites (chlorophyll *a*, chlorophyll *b*, total chlorophylls, carotenoids, anthocyanins, phenolic compounds) and the activity of enzymatic antioxidants (superoxide dismutase and guaiacol peroxide) in leaf explants varied depending on the AgNPs treatment and age of culture. Phenotype variations of ex vitro cultivated chrysanthemums, covering the color and pigment content in the inflorescence, were detected in one 50 mg·L^−1^ AgNPs-derived and five 100 mg·L^−1^ AgNPs-derived ‘Lilac Wonder’ plants and were manifested as the color change from pink to burgundy-gold. However, no changes in inflorescence color/shape were found among AgNPs-treated ‘Richmond’ chrysanthemums. On the other hand, the stem height, number of leaves, and chlorophyll content in leaves varied depending on the AgNPs treatment and the cultivar analyzed. A significant effect of AgNPs on the genetic variation occurrence was found. A nearly two-fold higher share of polymorphic products, in both cultivars studied, was generated by RAPD markers than by SCoTs. To conclude, protocols using leaf explant treatment with AgNPs can be used as a novel breeding technique in chrysanthemum. However, the individual cultivars may differ in biochemical response, the efficiency of in vitro regeneration, genetic variation, and frequency of induced mutations in flowering plants.

## 1. Introduction

Nanotechnology combines fundamental science, materials science, and engineering to design, characterize, and produce structures, devices, and systems by controlling the shape and size of materials at the nanometer scale [1]. The natural or manufactured nanomaterials are atomic or molecular aggregates, with unique and versatile physicochemical characteristics, e.g., high surface-to-volume ratio, increased catalytic activity, ability to engineer electron exchange, high reactivity, including interactions with living plant and animal cells, good electrical conductivity, or photochemical properties. These characteristics are different in comparison to material counterparts at the micrometric scale, which is directly related to the small size of nanomaterials, on a scale from 1 nm to 100 nm in one or more external dimensions or an internal structure, ensuring new applications of existing materials in various industrial sectors, biomedical sciences, pharmacology, daily life, agriculture, and biotechnology [2,3,4,5,6,7]. Silver nanoparticles (AgNPs) and gold nanoparticles (AuNPs) are the most often used nanomaterials in plant science, especially in agriculture and horticulture [5,6,7,8]. Nanomaterials have shown high potential for the development of new crop cultivars through genetic engineering or the production of efficient agrochemicals for plant nutrition and protection from pests. Nanotechnology also promotes the quality and enhancement of the shelf-life of non-processed and processed fruit, vegetables, and cut flowers [1,9,10,11]. Silver and gold nanoparticles applied in vitro at the concentration of 10 mg·L^−1^ increased the micropropagation efficiency in *Streptocarpus × hybridus* Voss. [12]. On the other hand, 10 mg·L^−1^ AuNPs significantly improved the cryopreservation efficiency in *Lamprocapnos spectabilis* (L.) Fukuhara [13]. In *Allium cepa* L., zinc oxide nanoparticles (ZnO NPs), in the concentration range from 50 to 1600 mg·L^−1^, stimulated the germination process of seeds [14].

Despite many perspectives and benefits arising from the enormous progress in nanotechnology development, nanomaterials at higher concentrations may also have adverse effects that have not been sufficiently explored during the implementation of the innovative agro-nanotechnologies in crop improvement. A good understanding of nanomaterials’ influence on plants at the morphological, histological, physiological, biochemical, and molecular levels is of paramount importance for assessing nanomaterials toxicity, evaluating environmental risks, food safety, and human health. The genotoxic effects, which are often difficult to predict, in particular, need deeper research [2,5,8]. After penetrating the plant cell wall, AgNPs can enter various organelles and cause cytotoxicity [8]. Patlolla et al. [15] demonstrated that AgNPs treatment resulted in an increased number of structural chromosomal aberrations, micronuclei induction, and a decreased value of the mitotic index in *Vicia faba* L. root meristem cells. The genotoxicity increased with the increasing concentration of AgNPs, from 12.5 to 100 mg·L^−1^. In another experiment, the 10, 20, 40, and 50 mg·L^−1^ AgNPs-treated root tip cells of *Triticum aestivum* L. exhibited various types of chromosomal aberrations [16].

The main mechanism underlying the AgNPs phytotoxicity is the overproduction of reactive oxygen species (ROS) leading to oxidative stress in plant cells, lipid peroxidation, damage to cell membranes, proteins, and DNA [7,8,15]. Moreover, silver ions (Ag^+^) released from AgNPs can interact chemically or physicochemically with nucleic acids and induce DNA damage [17]. However, plants inherently acquire some defense strategies to overcome the toxicity triggered by nanomaterials by activating various enzymatic and non-enzymatic defense systems, reducing the toxic effects of ROS [18]. The most often synthesized enzymatic antioxidants are superoxide dismutase (SOD), catalase (CAT), ascorbate peroxidase (APX), guaiacol peroxidase (GPOX), dehydroascorbate reductase (DHAR), and glutathione reductase (GR). Anthocyanins and carotenoids are non-enzymatic antioxidants whose function is to scavenge free radicals and chelate metals under stress conditions [7,8,13,19,20]. However, beyond a limit of stress factors, the internal detoxification mechanism also fails to overcome the toxicity and ultimately leads to the activity of apoptosis in plant cells [18] or mutations in plant genetic material [6].

*Chrysanthemum* × *grandiflorum* (Ramat.) Kitam. is one of the most economically important and favored floricultural crops worldwide, ranking second in the cut flower trade, following the rose. Chrysanthemums are mainly valued for their multi-colored inflorescences characterized by an exceptionally long flowering period [6]. Their inflorescences are also used in the medical, food, and beverage industries due to their nutritive and biologically active components. The market demand for cultivars with new inflorescence characteristics, improved stress tolerance, or quality attributes, is increasing annually, being a great challenge for chrysanthemum breeders [21,22].

The most frequently used chrysanthemum breeding methods are generative crossing and mutagenesis [22]. However, the application of crossing and selection, as well as more sophisticated methods of molecular breeding, including transgenic technology and genome editing, face limitations in this species due to the high levels of ploidy and heterozygosity [21,23]. Mutation breeding has been used by plant breeders worldwide since the discovery in the 1920s that random, heritable mutations could be induced in plants through irradiation or chemical treatments. Gamma-rays, X-rays, UV light, and heavy-ion beams, as physical mutagens, cause a combination of greater chromosome deletions and point mutations, whereas the most commonly used chemical mutagens (e.g., ethyl methanesulphonate EMS; N-methyl-N-nitrosourea MNU; 1-ethyl-1-nitrosourea ENU; natriumazide NaN_3_) almost exclusively cause single base substitutions [24,25]. In chrysanthemum, induced mutagenesis allows for the creation of new cultivars with changed color, shape, or size of inflorescence; plant/leaf architecture; or flowering earliness, in a relatively short time, during the cultivation and flowering of the first mutation generation. The mutagen treatment most often involves in-vitro-isolated explants, such as whole leaves, leaf petioles, internodes, inflorescence peduncles, ligulate florets, ovaries, callus tissue, suspensions of cells, or protoplasts. The in-vitro-regenerated adventitious shoots are then transferred to a greenhouse for further cultivation and phenotype evaluation. Plants presenting valuable and stable mutations are selected as potential new cultivars [22,23,26].

Mutation induction with X- or Gamma-rays is, unfortunately, associated with the use of specialized devices located in medical centers or scientific institutions. The application of nanoparticles as a new chemical mutagen added into/onto the culture medium could make chrysanthemum breeding relatively easy and be routinely performed in in vitro plant laboratories, without the need for sophisticated equipment used in the new breeding technologies (NBTs). Despite the first report on the genotype and phenotype variation in chrysanthemum as a result of adventitious shoots regeneration on internodes inoculated on media supplemented with 5, 10, and 20 mg·L^−1^ AgNPs [6], the survey of the scientific literature reveals no information about induced mutagenesis with the use of nanomaterials. Adventitious shoot regeneration is a fascinating process involving immense cell fate transition in callus and spatial reorganization of cell identities. It involves pluripotency acquisition and de novo shoot organogenesis linked with changes in the epigenetic status of a cell (e.g., histone modification), resulting in further changes in cell re-programing. The induction of adventitious shoot formation is affected by endogenous auxin synthesis in certain explant cells, followed by very temporary auxin accumulation, in parallel with histone hyperacetylation. These cell clusters serve as a basis for de novo shoot formation and, stimulated by exogenous cytokinin, develop into an adventitious shoot [23,26,27]. Application of nanomaterials can affect this process, although the exact mechanism of NP action in the plant cell is not fully understood [6,12].

This study aimed to analyze the biochemical activity of leaf explants cultured in vitro, evaluate the effectiveness of in vitro adventitious shoots regeneration, and identify genetic and phenotypic variation among ex-vitro-cultivated plants in *Chrysanthemum* × *grandiflorum* (Ramat.) Kitam. ‘Lilac Wonder’ and ‘Richmond’ cultivars, induced as a result of in vitro application of silver nanoparticles colloid at the concentration of 50 and 100 mg·L^−1^. The selected cultivars were used previously in breeding programs due to their genetic and phenotypic uniformity and non-chimeric structure [6,12,26]. The ‘Lilac Wonder’ cultivar is characterized by a pink, full semi-ball inflorescence, whereas the ‘Richmond’ cultivar inflorescence is purple-pink, full, and flat in shape. Both cultivars are usually cultivated in greenhouses for cut flowers with the standard method, i.e., one stem with a single inflorescence.

## 2. Results

### 2.1. In Vitro Culture—Course and Efficiency of Adventitious Organogenesis

**Figure 1:**
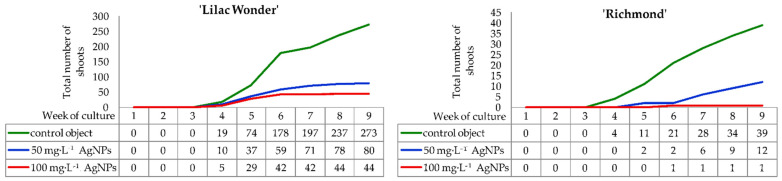
Dynamics of adventitious shoots regeneration on the inoculated *Chrysanthemum* × *grandiflorum* ‘Lilac Wonder’ and ‘Richmond’ leaf explants cultured on the modified MS medium with 0.6 mg·L^−1^ BAP and 2 mg·L^−1^ IAA, depending on the AgNPs treatment (0–100 mg·L^−1^).

In both chrysanthemum cultivars, the formation of light green callus on leaf petioles began in the second week of culture on control explants and in the third week on explants treated with silver nanoparticles (50 and 100 mg·L^−1^). Chrysanthemum ‘Lilac Wonder’ explants produced callus more intensively than ‘Richmond’ explants. In both cultivars tested, adventitious shoot regeneration occurred indirectly. The formation of the first adventitious shoots was observed in the fourth week of culture on ‘Lilac Wonder’ explants in all experimental combinations and on ‘Richmond’ control explants. Conversely, in ‘Richmond’, 50 mg·L^−1^ and 100 mg·L^−1^ AgNPs-treated leaves started to regenerate first shoots in the fifth and sixth week of culture, respectively (Figure 1).

In ‘Lilac Wonder’ chrysanthemum, the highest increase in the number of forming shoots was observed between the fourth and the sixth week of culture on the control medium and the media with 50 mg·L^−1^ and 100 mg·L^−1^ AgNPs. In the following weeks, the shoot regeneration dynamics were not as intensive, except for the control object, in which the number of shoots between the eighth and ninth week of culture increased by 36. Finally, with the control explants, the number of observed shoots in the ninth week was almost 273, while the numbers of shoots produced on explants treated with 50 mg·L^−1^ and 100 mg·L^−1^ AgNPs were only 80 and 44, respectively (Figure 1).

As for ‘Richmond’, successive and moderately intensive formation of shoots on the control explants continued until the end of culture and reached 39 shoots. Moreover, in this combination, the highest number of shoots was found, whereas only 12 shoots and 1 shoot were reported for 50 mg·L^−1^ and 100 mg·L^−1^ AgNPs treatments, respectively, in the ninth week. When silver nanoparticles were applied at the concentration of 50 mg·L^−1^, most of the shoots appeared between the sixth and ninth week of culture. In 100 mg·L^−1^ AgNPs treatment, the emergence of one shoot was noticed in the sixth week (Figure 1).

**Figure 2:**
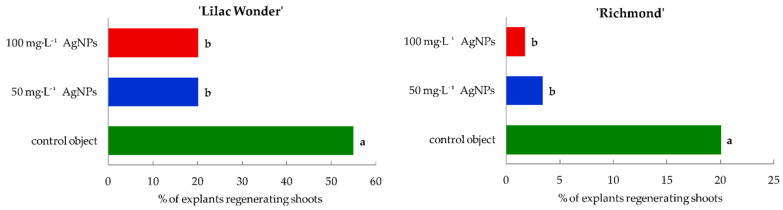
Shoot induction rate of *Chrysanthemum* × *grandiflorum* ‘Lilac Wonder’ and ‘Richmond’ leaf explants after 10 weeks of culture on the modified MS medium with 0.6 mg·L^−1^ BAP and 2 mg·L^−1^ IAA, depending on the AgNPs treatment (0–100 mg·L^−1^). Means on graphs for each cultivar tested followed by the same letter do not differ significantly at *p* ≤ 0.05 (Tukey’s test).

**Figure 3:**
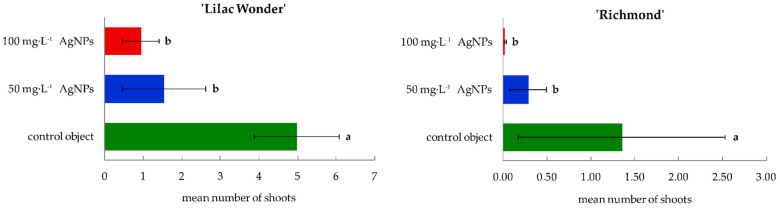
Mean number of adventitious shoots regenerated per one inoculated leaf explant in *Chrysanthemum* × *grandiflorum* ‘Lilac Wonder’ and ‘Richmond’ after 10 weeks of culture on the modified MS medium with 0.6 mg·L^−1^ BAP and 2 mg·L^−1^ IAA, depending on the AgNPs treatment (0–100 mg·L^−1^). Means ± SD on graphs for each cultivar tested followed by the same letter do not differ significantly at *p* ≤ 0.05 (Tukey’s test).

Silver nanoparticles treatment affected negatively the ability of leaf explants to form adventitious shoots and the efficiency of shoot regeneration, both in ‘Lilac Wonder’ and ‘Richmond’ cultivars. Moreover, differences between the cultivars tested in terms of the capability of adventitious organogenesis were observed (Figure 2, Figure 3 and Figure S1).

The shoot induction rate of ‘Lilac Wonder’ explants amounted to 55% in the control object and was reduced to 20% after the application of silver nanoparticles at the concentration of both 50 mg·L^−1^ and 100 mg·L^−1^. Similarly, AgNPs treatment also decreased the rate of ‘Richmond’ leaf explants forming shoots from 20% in the control object to only 1.67% in 100 mg·L^−1^ AgNPs treatment (Figure 2).

‘Lilac Wonder’ leaf explants treated with 50 and 100 mg·L^−1^ AgNPs regenerated significantly fewer shoots (1.53 and 0.92, respectively, per one explant inoculated), compared to non-treated explants forming, on average, almost 5 shoots. The same tendency resulting from silver nanoparticles application was observed in chrysanthemum ‘Richmond’ whose control leaf explants regenerated 1.35 shoots, whereas explants treated with 50 and 100 mg·L^−1^ AgNPs formed only 0.28 and 0.02 shoots, respectively (Figure 3).

### 2.2. In Vitro Culture—Biosynthesis of Metabolites and Enzymatic Activity of Leaf Explants

As for the results of chlorophyll *a*, chlorophyll *b*, total chlorophylls, and carotenoids extraction from in vitro cultured leaves, a general decrease in the content of these metabolites was observed starting from the first, through the second, and to the third week of culture, both in ‘Lilac Wonder’ and ‘Richmond’ cultivars, irrespectively of the AgNPs treatment. On the other hand, the AgNPs treatment significantly increased the content of chlorophyll *a*, chlorophyll *b*, and total chlorophylls in ‘Lilac Wonder’ explants, especially at 50 mg·L^−1^, while in ‘Richmond’, the highest tested concentration of AgNPs (100 mg·L^−1^) negatively affected the capability of explants to produce chlorophyll *b*, total chlorophylls, and carotenoids. As for the interaction between the AgNPs concentration and the week of culture, in ‘Lilac Wonder’, the highest content of chlorophyll *a*, chlorophyll *b*, total chlorophylls, and carotenoids was reported in the first week of culture for 0–100 mg·L^−1^ AgNPs treatments and in the second culture week for 50 mg·L^−1^ and 100 mg·L^−1^ AgNPs treatments, while the interaction between the control object and the third week of culture was characterized with the lowest content of these metabolites. Similarly, in ‘Richmond’, the highest values for chlorophyll *a*, chlorophyll *b*, total chlorophylls, and carotenoids contents were reported in the first week of culture, in the whole range of the tested AgNPs concentrations, and the second week of culture but for 0 mg·L^−1^ and 50 mg·L^−1^ AgNPs treatments. Explants treated with 100 mg·L^−1^ AgNPs and analyzed in the third week of culture had the lowest content of chlorophyll *a*, chlorophyll *b*, total chlorophylls, and carotenoids. No significant differences in the main effects and interactions between the tested experimental factors were observed for the values of chlorophyll *a*/*b* and chlorophylls/carotenoids ratios in ‘Richmond’, and chlorophylls/carotenoids ratio in ‘Lilac Wonder’. The highest chlorophyll *a*/*b* ratio was found in the third week of culture in 100 mg·L^−1^ AgNPs-treated ‘Lilac Wonder’ leaf explants (Table 1).

The content of anthocyanins in ‘Lilac Wonder’ leaf explants was stable; no significant differences were reported depending on the silver nanoparticles application or duration of the in vitro culture. Nevertheless, in ‘Richmond’, the most intensive biosynthesis of these pigments occurred in the first week of culture. The content of phenolic compounds in ‘Richmond’ leaf explants was not affected by the two tested experimental factors, unlike in ‘Lilac Wonder’. The concentration of phenolics in the latter cultivar was the lowest in the third week of culture compared to the first and the second week, irrespectively, and the biosynthesis of these compounds was mostly triggered by the 100 mg·L^−1^ AgNPs treatment (Table 1).

**Figure 4:**
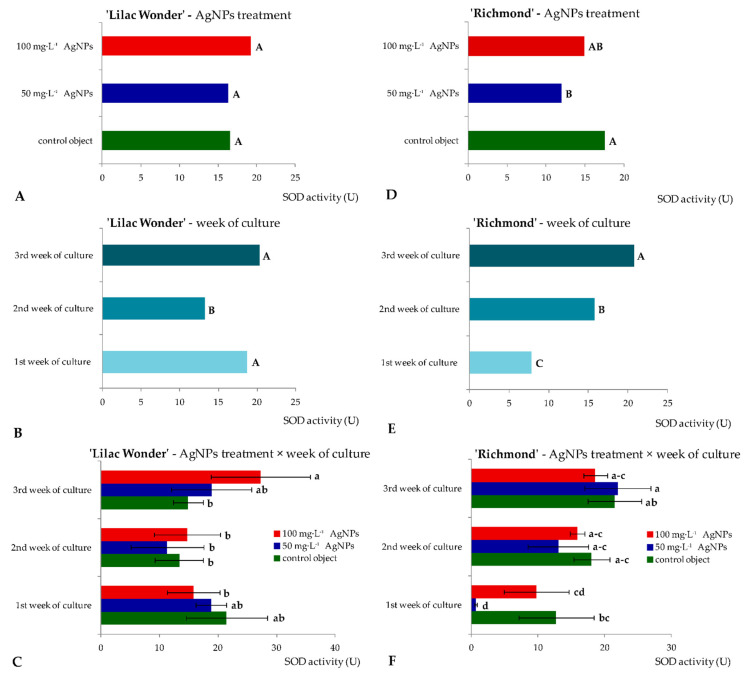
Activity of superoxide dismutase in *Chrysanthemum* × *grandiflorum* ‘Lilac Wonder’ (graphs **A**–**C**) and ‘Richmond’ (graphs **D**–**F**) leaf explants cultured in vitro on the modified MS medium with 0.6 mg·L^−1^ BAP and 2 mg·L^−1^ IAA, depending on the AgNPs treatment (0–100 mg·L^−1^) and week of culture (first–third). Means and means ± SD on graphs for each cultivar tested followed by the same letter do not differ significantly at *p* ≤ 0.05 (Tukey’s test). Upper-case letters refer to the main effects (irrespectively) (graphs **A**,**B**,**D**,**F**), lower-case letters refer to the interaction between the two studied independent variables (graphs **C**,**F**).

**Figure 5:**
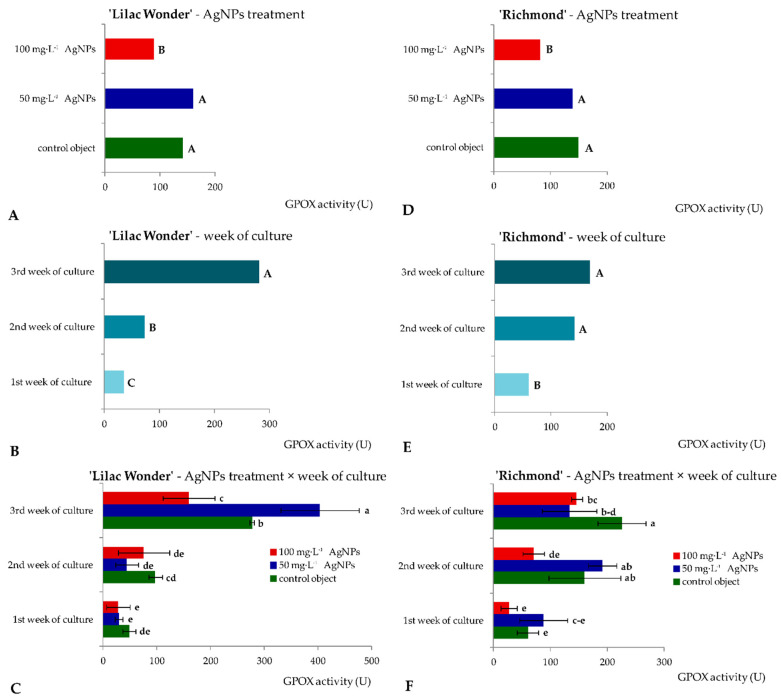
Activity of guaiacol peroxidase in *Chrysanthemum* × *grandiflorum* ‘Lilac Wonder’ (graphs **A**–**C**) and ‘Richmond’ (graphs **D**–**F**) leaf explants cultured in vitro on the modified MS medium with 0.6 mg·L^−1^ BAP and 2 mg·L^−1^ IAA, depending on the AgNPs treatment (0–100 mg·L^−1^) and week of culture (first–third). Means and means ± SD on graphs for each cultivar tested followed by the same letter do not differ significantly at *p* ≤ 0.05 (Tukey’s test). Upper-case letters refer to the main effects (irrespectively) (graphs **A**,**B**,**D**,**F**), lower-case letters refer to the interaction between the two studied independent variables (graphs **C**,**F**).

Similar levels of superoxide dismutase activity (16.33–19.25 U) in ‘Lilac Wonder’ leaf explants were observed depending on the silver nanoparticles treatment and irrespectively of the culture duration (Figure 4A). However, in ‘Richmond’, the highest and lowest SOD activity was found in the control and 50 mg·L^−1^ AgNPs-treated leaf explants (17.43 and 11.89 U, respectively) (Figure 4D). On the other hand, the lowest SOD activity was reported in the second week of culture in ‘Lilac Wonder’ (13.15 U), irrespectively of the AgNPs treatment (Figure 4B), whereas in ‘Richmond’, a significant increase in SOD activity in the successive culture weeks was observed (7.73–20.68 U) (Figure 4E). As for the interaction between the two tested factors, i.e., the AgNPs treatment and culture duration, the highest SOD activity was found in three-week-old and 100 mg·L^−1^ AgNPs-treated ‘Lilac Wonder’ leaf explants (27.25 U, Figure 4C) and in three-week-old and 50 mg·L^−1^ AgNPs-treated ‘Richmond’ leaf explants (21.96 U, Figure 4F). On the other hand, the application of silver nanoparticles at the highest tested concentration of 100 mg·L^−1^ significantly decreased the activity of guaiacol peroxidase, both in ‘Lilac Wonder’ and ‘Richmond’ leaf explants, irrespectively of the week of culture (Figure 5A,D). As for the influence of the culture duration, the lowest GPOX activity was found in the first week, both in ‘Lilac Wonder’ (35.02 U) and ‘Richmond’ (58.49 U) cultivars (Figure 5B,E). In ‘Lilac Wonder’, the activity of this enzyme was the highest for the interaction of 50 mg·L^−1^ AgNPs × third week of culture (403.46 U) and the lowest for the interaction of 100 mg·L^−1^ AgNPs × first week of culture (27.65 U) (Figure 5C). Considering the second tested cultivar, the interactions of 100 mg·L^−1^ AgNPs × first week of culture and control object × third culture week were characterized by the lowest and highest guaiacol peroxide activities, 27.38 U and 225.56 U, respectively (Figure 5F).

### 2.3. Phenotype Analysis of Ex Vitro Grown Plants

All ‘Lilac Wonder’ and ‘Richmond’ microshoots transferred onto the rooting medium were able to regenerate adventitious roots in two weeks, irrespective of nanoparticle treatment. Acclimatization was fully successful for the standard, 50 mg·L^−1^, and 100 mg·L^−1^ AgNPs-derived plants in ‘Richmond’ and also for all standard and 100 mg·L^−1^ AgNPs-treated ‘Lilac Wonder’ plants. As for the control and 50 mg·L^−1^ AgNPs-derived ‘Lilac Wonder’ chrysanthemums, 59% and 56.5% of them, respectively, survived the acclimatization process. Losses in the number of acclimatized plants were caused by fungal contamination and the weaker ability of plants to adapt to ex vitro conditions during acclimatization in the greenhouse. Flowering was not significantly influenced by AgNPs treatment and occurred in 74.6–96.0% ‘Lilac Wonder’ chrysanthemums and 68–100% ‘Richmond’ chrysanthemums (Table 2, Figure S2).

**Figure 6:**
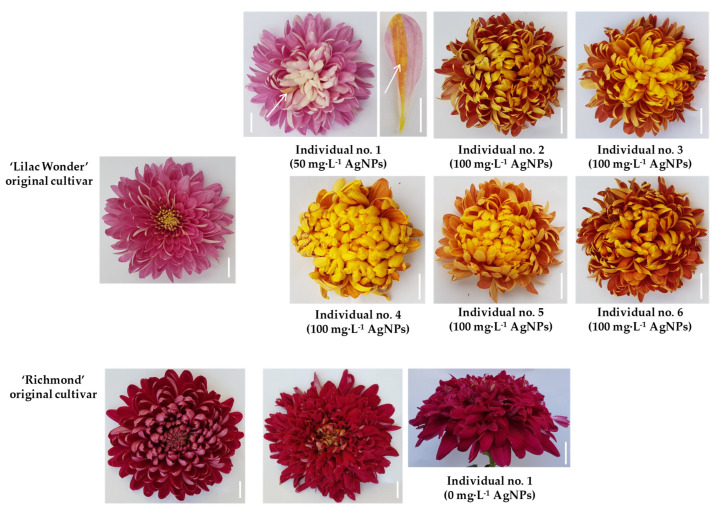
*Chrysanthemum× grandiflorum* ‘Lilac Wonder’ and ‘Richmond’ and their mutants, created as a result of AgNPs treatment (0–100 mg·L^−1^); arrows indicate a chimeric structure of the ligulate floret; bar = 1 cm.

All flowering ‘Lilac Wonder’ standard and control plants formed typical pink inflorescences. One mutant was identified among flowering 50 mg·L^−1^ AgNPs-derived plants (Individual No. 1). The mutation was phenotypically manifested by the change of inflorescence color—from pink to light pink. This plant was also of chimeric structure because part of one ligulate floret was covered with a narrow sector (stripe) of burgundy-gold color. Five mutants with changed color of the whole inflorescence were observed among 100 mg·L^−1^ AgNPs-treated chrysanthemums; the frequency of mutant/mutation occurrence amounted to 10%. Three plants were characterized by changed inflorescence color to burgundy-gold (Individuals No. 2, 3, and 6). The mutation identified in Individual No. 4 and Individual No. 5 was related to inflorescence color change to light burgundy-gold. No mutations concerning inflorescence shape were found among the flowering ‘Lilac Wonder’ chrysanthemums. On the other hand, all flowering ‘Richmond’ chrysanthemums formed typical purple-pink inflorescences, regardless of experimental treatment. However, the shape of one plant from the control treatment (0 mg·L^−1^ AgNPs; Individual No. 1) was changed from full and flat to full and irregular (Table 2 and Table 3, Figure 6). The observed inflorescence mutations were stable during the second cultivation and flowering cycle of mutated chrysanthemums.

The biochemical analysis of extracts prepared from ligulate florets of standard and mutated ‘Lilac Wonder’ chrysanthemums showed that inflorescence color changes resulted both from quantitative and qualitative differences in the content of pigments. Unlike the standard, inflorescences of all ‘Lilac Wonder’ mutants contained carotenoids. Ligulate florets of Individual No. 1 and Individual No. 2 were characterized by a lower content of anthocyanins compared to the standard, whereas in ligulate florets of Individuals No. 3–6, an increase in the content of anthocyanins was reported. No qualitative differences in pigment content were found between the inflorescences of ‘Richmond’ standard plants and the identified mutant (Table 3).

Results of biometric measurements of the developed plants at the full flowering stage revealed that ‘Lilac Wonder’ control and 50 mg·L^−1^ AgNPs-derived chrysanthemums developed the longest stems (54.79 and 56.09 cm, respectively) with the highest number of leaves (30.26 and 29.59, respectively). Nonetheless, chrysanthemums from these two experimental objects contained less chlorophyll in leaves (22.02–22.39 CCI) than the standard plants (22.97 CCI). No significant differences were found between the standard plants and 100 mg·L^−1^ AgNPs-derived plants in terms of the stem length, the number of leaves, and chlorophyll content. On the other hand, ‘Richmond’ plants regenerated on explants treated with silver nanoparticles at the concentration of 50 mg·L^−1^ produced the shortest stems (49.74 cm) during ex vitro cultivation as compared to the other treatments (61.91–70.00 cm). The highest number of leaves was reported on the stems of standard chrysanthemum (42.11) and 100 mg·L^−1^ AgNPs-derived plants (50). No differences were found in chlorophyll content depending on nanoparticles treatment. The size of inflorescence diameter was not affected by AgNPs application, both in ‘Lilac Wonder’ (7.02–7.59 cm) and in ‘Richmond’ (7.7–9.08 cm) (Figure S3).

### 2.4. Analysis of Genetic Fidelity of Ex Vitro Grown Plants

A total of 9563 scorable bands were detected by five RAPD and five SCoT primers in 153 plants (Table 4). Among the molecular marker systems tested, RAPDs generated more polymorphic products in both cultivars studied (28.6 and 51.7% mean per primer) than SCoTs (13.2 and 33.0%). A similar share of polymorphic plants was detected in the two studied cultivars by RAPD markers (28.2% in ‘Richmond’ and 26.5% in ‘Lilac Wonder’); however, a much higher number of genotypes (11–13) could be distinguished by both molecular marker systems in the ’Richmond’ plants compared to ‘Lilac Wonder’ (2–3). Primers R1 and S5 generated the highest number of bands in ‘Lilac Wonder’ (12 per sample). The former was also the most effective in screening for variation (all generated *loci* were polymorphic). As for the cultivar ‘Richmond’, the S1 primer produced 12 amplicons per sample. On the other hand, primer R5 generated only two amplicons in ‘Lilac Wonder’.

An over two-fold higher heterozygosity index (H) was reported in the ‘Richmond’ plants compared to ‘Lilac Wonder’, according to both RAPD and SCoT markers (Table 5). A similar tendency could also be found with the other polymorphic indices, except for the resolving power (R) of RAPD markers, which was higher in ‘Lilac Wonder’. Among the two marker systems used, higher mean PIC, D, and R values were detected by RAPDs in both cultivars studied. Only in the case of the H, E, and MI indices were higher values detected in ‘Richmond’ chrysanthemums by SCoTs.

**Figure 7:**
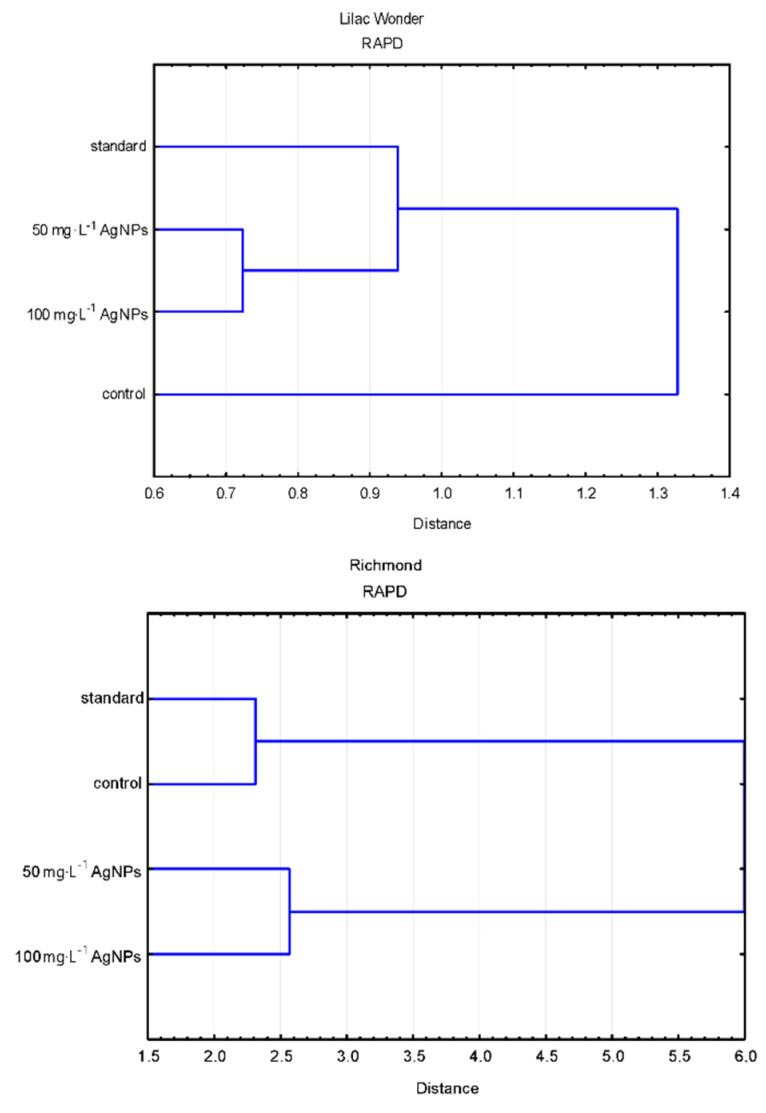

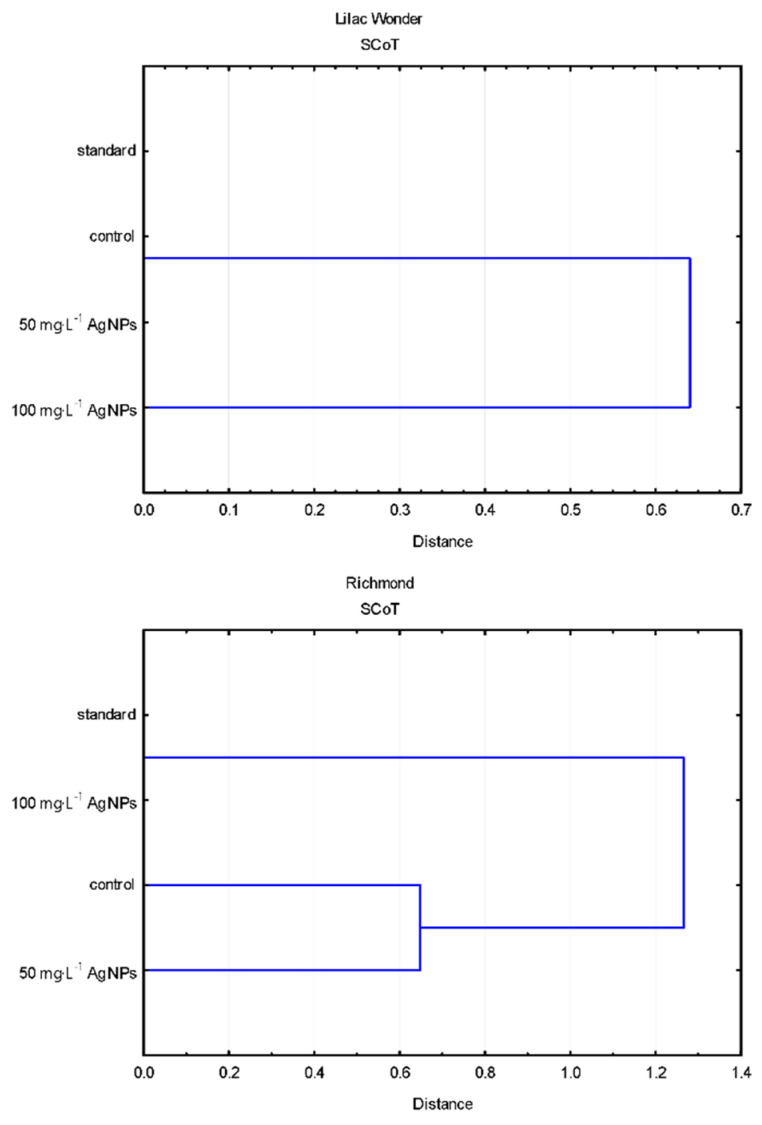
Dendrograms based on the estimation of genetic distance coefficient and UPGMA clustering present in the relationships between the populations of AgNPs-treated, control, and standard plants, revealed by the randomly amplified polymorphic DNA (RAPD) and start codon targeted polymorphism (SCoT) analyses.

Polymorphic plants could be found in the untreated control plants (0 mg·L^−1^) after 50 mg·L^−1^ AgNPs treatment, 100 mg·L^−1^ AgNPs treatment (only in ‘Lilac Wonder’), and in the standard plants of ‘Lilac Wonder’. The results of the grouping UPGMA analysis of the studied populations varied depending on the marker system used (Figure 7).

As for ‘Lilac Wonder’, according to the RAPD analysis, the control population derived from adventitious shoots not treated with AgNPs (0 mg·L^−1^) was the most distant from the remaining populations placed in a single cluster divided into two sub-clusters (standard and AgNPs-treated). On the other hand, according to the SCoT analysis, the population of chrysanthemums treated with 100 mg·L^−1^ AgNPs was significantly different from the other three populations grouped in a single cluster (Figure 7).

According to the RAPD analysis of the cultivar ‘Richmond’, two clusters could be distinguished. The first cluster comprised the control and standard populations, while the second cluster contained AgNPs-treated plants. As for the SCoT fingerprinting, two clusters were also recognized, but the first one included the standard and 100 mg·L^−1^ AgNPs-derived plants, while the other—control and 50 mg·L^−1^ AgNPs-treated populations (Figure 7).

**Figure 8:**
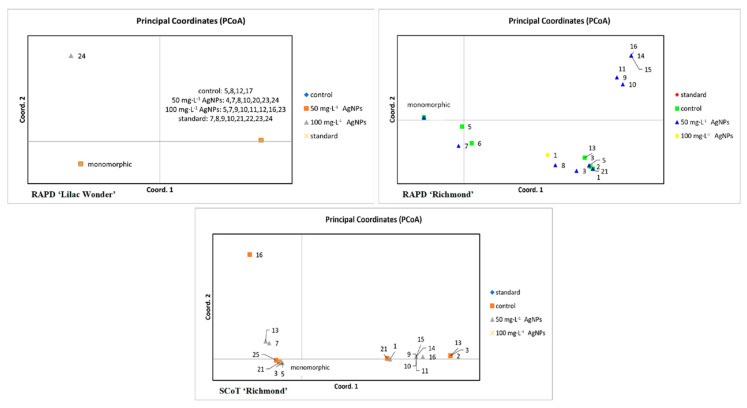
Graphs of principal coordinates analysis (PCoA) of *Chrysanthemum* × *grandiflorum* ‘Lilac Wonder’ and ‘Richmond’ plants obtained after 0 (control), 50, and 100 mg·L^−1^ AgNPs treatment and in standard plants, based on randomly amplified polymorphic DNA (RAPD) and start codon targeted polymorphism (SCoT) analyses. Plants representing the same band pattern as the predominant standard are collected within a single group named ‘monomorphic’.

A slightly different interpretation of the results was possible with the PCoA analysis of individual plants (Figure 8). According to the RAPD analysis of ‘Lilac Wonder’, a single genotype obtained after 100 mg·L^−1^ AgNPs treatment was significantly different from the remaining plants arranged into two groups. However, due to the low polymorphism level detected only in a single plant from the 100 mg·L^−1^ AgNPs object, no group differentiation was possible with the SCoT markers.

As for the cultivar ‘Richmond’, four groups were distinguished by the RAPDs, with five plants (representing three genotypes) obtained as a result of 50 mg·L^−1^ AgNPs treatment being the most distant from the predominant standard. Based on the SCoT analysis, three major groups of plants could be distinguished, with six genotypes (12 plants) from the control and 50 mg·L^−1^ AgNPs objects significantly different from the standard.

The AMOVA analysis confirmed a significant influence of the experimental treatments on the occurrence of interspecific genetic variation in most experimental objects (reaching up to 32% of the total variation in ‘Richmond’), except for the SCoT analysis in chrysanthemum ‘Lilac Wonder’ (Φ_PT_ = −0.029; Table S1).

## 3. Discussion

### 3.1. Effect of Silver Nanoparticles on the Regeneration Efficiency in Leaf Explants

Micropropagation through adventitious shoots regeneration from non-meristematic explants combined with mutagen treatment is a commonly exploited technique in chrysanthemum breeding, allowing for a restitution, from a single mutated explant cell, of genetically homogenous plants with a changed phenotype. The application of mutagens usually limits the regeneration capacity of the treated explants [23,26]. For example, γ-radiation at the dose of 15 Gy limited the formation of adventitious shoots on internodes in ‘Satibleu’ chrysanthemum and inhibited it completely on leaf explants in ‘Albugo’ and ‘Satinbleu’ [23]. In the present study, silver nanoparticles decreased the shoot induction rate of leaf explants and the number of formed shoots, both in ‘Lilac Wonder’ and ‘Richmond’ cultivars. Similarly, in the study by Tymoszuk and Miler [12], treatment with AgNPs at the concentration of 50 mg·L^−1^ and 100 mg·L^−1^ reduced the shoot induction rate of internodes from 90% in the control to 42% and 16%, respectively, in ‘Lilac Wonder’, and from 68% in the control to 24% and 12%, respectively, in ‘Richmond’. The control explants of chrysanthemum ‘Lilac Wonder’ produced, on average, 8.4 shoots, while internodes treated with 50 mg·L^−1^ and 100 mg·L^−1^ regenerated only 1.34 and 0.92 shoots, respectively. The same tendency was observed in ‘Richmond’ internodes treated with nanoparticles (3.22 shoots in control versus 0.88 shoot at 50 mg·L^−1^ AgNPs and 0.40 shoot at 100 mg·L^−1^ AgNPs). The regeneration of adventitious shoots is related to the acquisition of pluripotency by the callus cells and their further dedifferentiation and redifferentiation [23,26]. This phenomenon is manifested by a change in the cell shape, gene expression pattern, protein expression pattern, and function [27]. Apparently, in the present study, AgNPs precluded cell re-programing and stabilized cell identity, possibly by preventing temporary stress induction. It is generally known in mutation breeding that the regeneration capacity is negatively correlated with irradiation dose. However, with the decrease in regeneration capacity, the mutation frequency increases [22]. Even though the mutagenic factors, either physical or chemical, modulate the course and efficiency of adventitious shoots organogenesis, a considerable influence of the genotype tested and explant type in this process can be observed. Chrysanthemum cultivars differ in the efficiency of regeneration, and internodes seem to be more efficient in terms of adventitious shoot proliferation. Perhaps it would be more effective to apply silver nanoparticles on leaf explants with a previously proliferated callus on petioles than to use excised leaves for direct treatment with AgNPs. Such an approach was more effective in mutation induction with γ-radiation and microwaves in ‘Alchimist’ chrysanthemum, as reported by Zalewska et al. [23] and Miler and Kulus [29].

### 3.2. Effect of Silver Nanoparticles on the Biochemical Events in Leaf Explants

Similarly to our study, variable results concerning the response of plant cells at the biochemical level after AgNPs treatment were also reported earlier in chrysanthemum ‘Lilac Wonder’ calli and adventitious shoots regenerated on 10-week-old internodes cultivated on media supplemented with 5 mg·L^−1^, 10 mg·L^−1^, or 20 mg·L^−1^ AgNPs. In calli, the concentration of chlorophylls and polyphenols was stable, regardless of AgNPs concentration, but the production of carotenoids was enhanced with 20 mg·L^−1^ AgNPs-treatment. In contrast, in shoots, the content of chlorophylls was lower with 10 mg·L^−1^ and 20 mg·L^−1^ treatments, but no differences in carotenoids and phenolic compounds were found [6]. Usually, the increase in carotenoids content (which act as antioxidants) and the decline in chlorophylls content (which are the most unstable plant pigments) are associated with oxidative stress events in the cells induced by AgNPs [3,6,30]. Moreover, polyphenols and anthocyanins are markers of oxidative stress in plants, and their enhanced accumulation is often induced by nanoparticles treatment [3]. Nevertheless, the response related to phenolics biosynthesis of individual genotypes may be short and reversible [31] and usually induced when nanoparticles are applied at very high concentrations [32]. To summarize, the biochemical response of plant cells related to the biosynthesis of primary and secondary metabolites definitely depends on the nanoparticles treatment, but the changes occurring in the profile of individual compounds are not only genotype specific but also depend on the period between NPs treatment and performance of biochemical analysis, as well as the plant tissue/organ type selected for the analysis.

Studies on gold nanoparticles (10–30 mg·L^−1^) application during different stages of a cryopreservation protocol in *Lamprocapnos spectabilis* ‘Valentine’ revealed that the activities of SOD, APX, and CAT were significantly higher when AuNPs were added at higher concentration into the preculture (SOD) or recovery media (APX, CAT), but no differences were found in GPOX activity [13]. As for *Solanum lycopersicum* L. ‘Poranek’, *Raphanus sativus* L. var. *sativus* ‘Ramona’, and *Brassica oleracea* var. *sabellica* ‘Nero di Toscana’, the three-week-old seedlings developed from seeds treated with 50 and 100 mg·L^−1^ AgNPs did not differ in terms of SOD activity. The 100 mg·L^−1^ AgNPs treatment resulted in an almost two-fold higher GPOX activity in tomato but decreased the activity of this enzyme in radish. As for kale, a significant increase in GPOX activity was found with both 50 and 100 mg·L^−1^ AgNPs concentrations. These results are in line with our findings and clearly show that nanoparticles interact differentially with individual plant species and that elevation/reduction in activity or inactivation of particular enzymes can occur over time [7].

### 3.3. Effect of Silver Nanoparticles on the Phenotype Stability

In the present experiment, ‘Lilac Wonder’ cultivar treated with 50 mg·L^−1^ AgNPs produced one plant with a changed inflorescence color from pink to light pink and with a chimeric ligulate floret with a sector of burgundy-gold color. Among 100 mg·L^−1^ AgNPs-derived ‘Lilac Wonder’ chrysanthemums, three mutants with changed inflorescence color to burgundy-gold and two mutants with light burgundy-gold inflorescence were identified. The burgundy-gold chrysanthemum cultivars are rare and, at the same time, desirable as a breeding goal for the horticultural market. No alternations of inflorescence color/shape were found among AgNPs-derived ‘Richmond’ chrysanthemums. ‘Lilac Wonder’ cultivar was also used in another study, in which a different explant type (internodes) was tested, and a different method of NPs application was used; AgNPs were added into a regeneration medium at lower concentrations of 5 mg·L^−1^, 10 mg·L^−1^, and 20 mg·L^−1^ [6]. Consequently, one mutant with a pink-gold inflorescence was found after the application of 10 mg·L^−1^ AgNPs, while three mutants with light pink inflorescence, one mutant with burgundy-gold, and one mutant with dark pink inflorescences were identified among 20 mg·L^−1^ AgNPs-derived plants. Moreover, one of the light pink mutants presented a changed inflorescence type, and the second light pink mutant was of chimeric structure with a red stripe on one ligulate floret. The frequency of mutant/mutations occurrence amounted to 1%/1% for 10 mg·L^−1^ and 6.3%/8.9% for 20 mg·L^−1^, respectively, and it was lower than in the present study with the higher AgNPs concentrations tested (2.3%/2.3% at 50 mg·L^−1^ and 10%/10% at 100 mg·L^−1^). This comparison shows that in the same cultivar tested, different results in mutation induction can be obtained depending on the explant type used, AgNPs concentration, and the method of their application. One should also keep in mind that mutation induction is a random phenomenon and can also occur spontaneously during the in vitro and/or ex vitro chrysanthemum growth, as in the present study, among flowering control shoots in ‘Richmond’.

In chrysanthemum breeding with the use of γ-radiation, white-flowering chrysanthemum ‘Albugo’ produced eight mutants with a yellow inflorescence, while dark pink ‘Satinbleu’ produced six mutants of pale pink inflorescences [23]. The appearance of identically changed chrysanthemum mutants, in terms of inflorescence color, was also reported by Zalewska [33] and explained as the existence of some genotype-specific variation trends. On the other hand, Broertjes et al. [34] pointed to the possibility of a multi-meristem formation from the initial mutated cell and, as a consequence, the growth of more than one adventitious shoot from the same adventitious meristemoid, although it is generally accepted that adventitious shoot formation occurs from a single cell in chrysanthemum [35] and in many other plant species [36].

The spectrophotometric analysis of pigments in ligulate florets of mutants obtained in the present study revealed that variations of inflorescence color were a result of both quantitative and qualitative differences in the content of anthocyanins and carotenoids. These findings are in agreement with those reported for AgNPs-derived mutants in ‘Lilac Wonder’ [6] and for ‘Albugo’, ‘Alchimist’, and ‘Satinbleu’ mutants created as a result of γ-irradiation [23]. It is worth emphasizing that each radiomutant, compared to its original cultivar, presents its own permanent and repetitive profile of specific inflorescence pigments, which gives a possibility of showing the distinctness and identification of a new cultivar through chemotaxonomy [37,38]. The control and 50 mg·L^−1^ AgNPs-derived flowering ‘Lilac Wonder’ chrysanthemums had the longest stems compared to standard plants. However, 50 mg·L^−1^ AgNPs-derived ‘Richmond’ chrysanthemums developed the shortest stems. No AgNPs influence on the inflorescence diameter, both in ‘Lilac Wonder’ and in ‘Richmond’, was found. Miler and Kulus [29] reported that ‘Alchimist’ chrysanthemums regenerated from microwave-treated leaf explants (2, 4, 6, and 8 s at 2.45 GHz, 800 W·cm^2^) produced longer shoots (by 39%) and inflorescences of greater diameter (by 21.5%) compared to the control. An interesting effect of enhanced growth, stimulated by a low dose of irradiation, was observed in ‘Profesor Jerzy’ and ‘Karolina’ chrysanthemum cultivars. Plants originating from ovaries irradiated with 5 Gy high-energy photons had the longest stems; 10 Gy-derived plants were medium sized, while 15 Gy-derived plants were the shortest [22]. Such dependences observed in the biometrical characteristics of plants most likely arise not only from the nanoparticles/microwaves/ionizing radiation treatment and dose of mutagenic factor but are also related to cultivar specificity or the explant type used.

### 3.4. Effect of Silver Nanoparticles on the Genetic Stability

In the present study, a nearly two-fold higher share of polymorphic products, in both cultivars studied, was generated by RAPD markers than by SCoTs. Our findings are in agreement with those reported by Lema-Rumińska et al. [39] and Kulus et al. [40], who also described RAPDs as the most effective in screening for variation in chrysanthemum and other members of the Asteraceae botanical family compared to the inter-simple sequence repeat (ISSR) system. Therefore, RAPDs can still be considered an inexpensive yet powerful typing method for plants.

The results of the AMOVA analysis showed a significant influence of AgNPs on the genetic variation occurrence during in vitro adventitious organogenesis from leaf explants in chrysanthemum. A similar phenomenon was reported by Tymoszuk and Kulus [6] in the adventitious shoots of ‘Lilac Wonder’ cultivar regenerated from internode explants. However, in the present study, a much higher share of polymorphic plants (up to 28.2%) was observed compared to the previous research (11.4%). This is probably due to the five-fold higher concentration of AgNPs used (50–100 mg·L^−1^). Our findings confirm the genotoxic character of nanoparticles, also described by other authors [41], particularly if applied at a high concentration. It can also be suggested that AgNPs have a higher mutagenic potential than the gold nanoparticles that induced variation only in 7.5% of *Lamprocapnos spectabilis* (L.) Fukuhara plants at 100 mg·L^−1^ concentration [42].

Interestingly, a significant effect of the genotype factor on the efficiency of mutation induction was observed. According to both marker systems analyzed, chrysanthemum ‘Richmond’ generated significantly more new genotypes than ‘Lilac Wonder’. This cultivar was also characterized by higher values of nearly all polymorphic indices described in Table 5. The diverse efficacy of individual chrysanthemum cultivars in mutation breeding was previously described by Schum [43]. Chrysanthemum ‘Richmond’ was used by Jerzy and Zalewska [44] to create several other cultivars of the ‘Lady’ group through X- and γ-radiation. Apparently, this cultivar has more hot spots in the genome, with a high frequency of mutation occurrence, than ‘Lilac Wonder’ [45]. However, the lack of evident phenotypical alternations in ‘Richmond’ cultivar suggests that the induced changes in the DNA sequence occurred in the non-coding parts of the genome. In future studies, the size of mutations induced by nanoparticles could be assayed with the genomic in situ hybridization (GISH) technique [46].

## 4. Materials and Methods

### 4.1. Plant Material—In Vitro Culture Conditions and Nanoparticles Treatment

For in vitro culture, the modified MS medium [47] was used. The content of calcium and iron in the medium was increased by half. The medium contained 30 g·L^−1^ sucrose and 8 g·L^−1^ Plant Propagation LAB-AGAR^TM^ (BIOCORP, Warsaw, Poland). The medium was supplemented with plant growth regulators (PGRs): 0.6 mg∙L^−1^ 6-benzylaminopurine (BAP) and 2 mg∙L^−1^ indole-3-acetic acid (IAA) (Sigma-Aldrich, St. Louis, MO, USA) to stimulate the regeneration of adventitious shoots. The medium pH was adjusted to 5.8. Next, 40 mL of the medium was poured into 350 mL glass jars sealed with plastic and autoclaved at 105 kPa and 121 °C for 20 min.

Leaves, dissected from plantlets cloned by the single-node method on the modified MS medium without PGRs or AgNPs, were used as explants. Four leaves were placed in a vertical position per each culture jar filled with the medium for adventitious shoots regeneration. Immediately after inoculation in the medium, the explants were treated with silver nanoparticles at the concentration of 50 and 100 mg·L^−1^ (0.05 and 0.1 mg∙mL^−1^, respectively). The AgNPs solutions were sterilized with the use of syringe filters (Minisart^®^ RC 25, pore size 0.20 µm; Sartorius AG, Göttingen, Germany). Next, the nanocolloids were poured onto the culture medium—2 mL of AgNPs colloid at each tested concentration per culture jar. Explants inoculated on the regeneration medium without AgNPs (0 mg·L^−1^) were used as the control. Silver nanoparticles were manufactured by Nanoparticles Innovation NPIN s.c. (Łódź, Poland). The AgNPs were produced by the seeded-mediated growth method [48,49] and characterized by the hydrodynamic size in colloids reaching 23 ± 4 nm (dynamic light scattering, DLS; Nano ZS Zetasizer system, Malvern Instruments, Malvern, UK). The size and size distribution measured by scanning transmission electron microscopy (STEM) (Nova NanoSEM 450, FEI^TM^, Hillsboro, OR, USA) at accelerating voltage 30 kV were 20 ± 3 nm. The DLS size distribution graphs and STEM images of the used silver nanoparticles are presented in Tymoszuk and Miler [12] and Tymoszuk and Kulus [6].

In vitro cultures were incubated in the growth room at a constant temperature of 23 ± 1 °C, under a 16 h photoperiod, using Philips TLD 36W/54 fluorescent lamps emitting cool daylight (Koninklijke Philips Electronics N.V., Eindhoven, the Netherlands). The photosynthetic photon flux density was set at 35 μmol m^−2^·s^−1^.

Observations of the dynamics of adventitious shoots regeneration on all inoculated leaf explants depending on the experimental treatment were performed weekly for nine successive weeks. In the 10th culture week, the shoot induction rate of explants and the mean number of shoots per one inoculated explant were estimated.

Regenerated adventitious shoots (2–3 cm in length, non-hyperhydrated, in a maximum number of 100 from each experimental object) were cut off from leaf explants and transferred onto the modified MS rooting medium supplemented with 2.0 mg·L^−1^ IAA for two weeks. Additionally, 25 shoots multiplied in vitro via the single-node method on the modified MS medium without PGRs and AgNPs were rooted for further ex vitro cultivation to form a genotype/phenotype standard of ‘Lilac Wonder’ and ‘Richmond’ plants.

### 4.2. Plant Material—Biochemical Array during In Vitro Culture

In the first, second, and third weeks of the in vitro culture, biochemical analyses were performed to assess the oxidative stress response of leaf explants after the application of 0, 50, and 100 mg·L^−1^ AgNPs. The whole-leaf explants were used as fresh tissue samples. Chlorophylls and carotenoids were extracted using 100 mg samples and 100% acetone (Chemia, Bydgoszcz, Poland) according to Lichtenthaler’s [50] procedure. Total anthocyanins with cyanidin-3-glucoside used as a standard were extracted using 200 mg samples and methanol containing 1% HCl (*v/v*) (Chemia, Bydgoszcz, Poland), as described by Harborne [51]. The same extract was used for the analysis of the total phenolic content according to the Folin–Ciocalteau protocol [52] with gallic acid (Sigma-Aldrich, St. Louis, MO, USA) as the calibration standard.

To determine the enzymatic activity, samples (100 mg) were prepared as described by Homaee and Ehsanpour [53]. The obtained extracts were used for the determination of the total protein content [54] and the activities of specific antioxidant enzymes. The superoxide dismutase (SOD; EC 1.15.1.1) activity was determined following the protocol elaborated by Giannopolitis and Ries [55]. The guaiacol peroxidase (GPOX; EC 1.11.1.7) activity was measured according to the Maehly and Chance [56] methodology, with modifications [57].

The spectrophotometric analyses were performed using the SmartSpec PlusTM spectrophotometer (BioRad, Hercules, CA, USA) at specific wavelengths (λ_max_): for anthocyanins (cyanidin-3-glucoside)—at 530 nm; for chlorophyll *a* and *b*—at 645 and 662 nm; for carotenoids—at 470 nm; for phenolics—at 765 nm; for proteins—at 595 nm; for SOD—at 560 nm; and for GPOX—at 470, respectively. The contents of the plant pigments and the phenolic compounds were calculated per 1 g of sample fresh weight (FW). The enzymatic activity U (μmol·min^−1^) was calculated per 1 mg of protein.

### 4.3. Plant Material—Acclimatization, Ex Vitro Growth, and Phenotype Analysis

Rooted plantlets were acclimatized in a glasshouse, in June, in natural light conditions, for two weeks. They were planted in plastic trays filled with a mixture of peat substrate (Hartman, Poznań, Poland) and perlite (Perlit, Šenov u Nového Jičína, Chech Republic) (2:1, *v*/*v*). Plants were regularly sprayed with water and covered with perforated transparent foil and geo-cover. Next, the plants were transferred to plastic pots (ø 24 cm, three plants per one pot) filled with peat substrate (Hartman, Poznań, Poland). In the early growing period, plants were cultivated vegetatively from July to mid-August in the natural photoperiod to achieve the shoot height of about 20 cm and develop the appropriate number of leaves. Starting from 15 August, further cultivation was conducted in 10 h short-day conditions to induce the generative development and bring plants to full flowering (dark phase, 6 PM–8 AM).

At the full flowering stage, the mutants (plants with changed morphological traits) and mutations (type of altered traits) were identified. The frequency of mutant and mutation occurrence was determined against the total number of flowering plants. The mutants were distinguished by defining the color and shape of the inflorescences of the standard (produced from nodal segments), control (0 mg·L^−1^ AgNPs), and AgNPs-treated plants. The color of the inner and outer sides of ligulate florets of fully developed inflorescences was established using the Royal Horticultural Society Colour Chart catalog (RHSCC) [28]. Moreover, all flowering plants were biometrically evaluated. Stem height (cm), number of leaves on the stem, and inflorescence diameter (cm) were measured. The content of chlorophyll in leaves was estimated with the use of a portable Chlorophyll Content Meter CCM-200 plus (Opti-Sciences, Hudson, NH, USA) and expressed in relative chlorophyll content index units (CCI). Moreover, the analysis of pigments occurrence and content in ligulate florets in the standard and mutant plants was performed. Carotenoids and total anthocyanins were extracted according to Lichtenthaler’s [50] and Harborne’s [51] protocols, respectively, as described earlier. The selected mutant plants were vegetatively propagated by stem cuttings and tested for inflorescence mutation stability during the second flowering stage.

### 4.4. Plant Material—Analysis of Genetic Stability of Ex Vitro Grown Plants

The genetic fidelity of AgNPs-treated plants was assessed using randomly amplified polymorphic DNA (RAPD) [58] and start codon targeted polymorphism (SCoT) [59] marker systems, during the full flowering stage.

Total genomic DNA was extracted from fresh leaf tissues. The Genomic Mini AX Plant SPIN Kit (A&A Biotechnology, Gdynia, Poland) reagents and materials were used to isolate DNA. The concentration of DNA was measured and standardized with the NanoPhotometer^®^ NP80 (Implen, München, Germany). The isolated DNA was stored at 4 °C.

The DNA samples were used as a template for the PCR analysis with a total of 10 primers (5 RAPD and 5 SCoT). PCR was performed in the BioRad C1000 Touch thermal cycler (Bio-Rad, Hercules, CA, USA) in the 25 µL reaction solution. The composition of the reaction solution, PCR profiles, and electrophoretic separation of amplified DNA fragments were described in detail in Lema-Rumińska et al. [39]. The PCR products were visualized on a UV transilluminator (GelDoc XR+ Gel Photodocumentation System with Image Lab 4.1 software, Bio-Rad, Hercules, CA, USA) after staining with ethidium bromide. The Gene Ruler^TM^ Express DNA Ladder (Thermo Fisher Scientific, Waltham, MA, USA) 100–5000 bp DNA marker was used as a size reference.

The banding patterns were recorded as 0–1 binary matrices, where ‘1’ indicates the presence and ‘0’ the absence of a given fragment followed by statistical analysis. For every primer tested, the numbers of monomorphic, polymorphic, and specific/unique *loci* were counted. Values of heterozygosity index (H), polymorphic information content (PIC), effective multiplex ratio (E), marker index (MI), discriminating power (D), and resolving power (R) were investigated for every primer and marker system used, independently for each cultivar [60].

### 4.5. Statistical Analysis

The experiment was set up in a completely randomized design. Each treatment in the experiment with in vitro cultures consisted of 15 jars (60 explants in total). One explant was considered as one repetition. All biochemical analyses performed at the in vitro and ex vitro stages were repeated six times. The obtained data were presented as mean ± standard deviation (SD) and subjected to a one-way or two-way analysis of variance (ANOVA) and post hoc Tukey’s test at the significance level of *p* ≤ 0.05. For data expressed as a percentage, the Freeman–Tukey double-arcsine transformation was used. Tables with results provide numerical data, with the alphabet indicating the homogeneous groups. All statistical analyses were performed with the use of the Statistica 13.3 software (StatSoft Polska, Cracow, Poland).

A total of 75 ‘Lilac Wonder’ plants (25 from 0 mg·L^−1^; 25 from 50 mg·L^−1^; and 25 from 100 mg·L^−1^ AgNPs treatment, respectively) and 43 ‘Richmond’ plants (25 from 0 mg·L^−1^; 17 from 50 mg·L^−1^; and 1 from 100 mg·L^−1^ AgNPs treatment, respectively), as well as 25 standard plants of each cultivar were included in the genetic stability analysis. The coefficient of genetic distance based on the Nei and Li algorithm [61] was calculated by a comparison of the predominant band pattern of the standard plants with the band patterns of the adventitious shoots regenerated from the leaf explants non-treated (0 mg·L^−1^; control) and treated with AgNPs (50 and 100 mg·L^−1^). The dendrograms were created based on agglomerative hierarchical clustering (AHC) with the unweighted pair group average method (UPGMA) using Statistica 13.3. Population groups were distinguished based on the analysis of molecular variance (AMOVA) and principal cluster analysis (PCoA) estimates using GeneAlEx 6.5 software [62] with the assumption that AgNPs-treated, control, and standard plants are three separate populations. iMEC software was used to calculate the polymorphism indices [60].

## 5. Conclusions

The results of the conducted experiment provide a better understanding of the multifaced influence of AgNPs on plants at the biochemical, genetic, and phenotypic levels. Silver nanoparticles applied at the concentrations of 50 mg·L^−1^ and 100 mg·L^−1^ limited the capacity of leaf explants to form adventitious shoots, affected the biosynthesis of primary and secondary metabolites, and modulated the activity of antioxidant enzymatic defense system in the tested chrysanthemum cultivars, similarly to the action of mutagens used in plant breeding but in a cultivar-specific way. The impact of AgNPs on the genetic variation occurrence during in vitro adventitious shoot organogenesis in the two tested cultivars and the later identification of mutations in the plants’ phenotype during ex vitro cultivation in ‘Lilac Wonder’ allow concluding that the proposed innovative and, at the same time, relatively easy-to-perform method of AgNPs use for mutation induction can find practical application in chrysanthemum breeding. Nevertheless, the response of individual cultivars to AgNPs treatment varies, and the changes induced at the genetic level are not always manifested in the phenotypic traits of chrysanthemum inflorescence or plant architecture during the final stage of cultivation. Future studies could focus on the use of NPs of a different type, size, and concentration to evaluate their mutagenic potential in different chrysanthemum cultivars or plant species and implement other methods for the assessment of cyto- and genotoxic effects of nanoparticles on plant cells.

## Figures and Tables

**Table 1:** Content of primary (chlorophyll *a*, chlorophyll *b*) and secondary (carotenoids, anthocyanins, phenolic compounds) metabolites in *Chrysanthemum* × *grandiflorum* ‘Lilac Wonder’ and ‘Richmond’ leaf explants cultured in vitro on the modified MS medium with 0.6 mg·L^−1^ BAP and 2 mg·L^−1^ IAA, depending on the AgNPs treatment (0–100 mg·L^−1^) and week of culture (first–third).

Concentration of AgNPs (A)	‘Lilac Wonder’				‘Richmond’			
	Week of Culture (B)							
	First	Second	Third	Mean	First	Second	Third	Mean
	Chlorophyll *a* content (mg·g^−1^ FW)							
**control object**	0.83 ± 0.11 ab	0.50 ± 0.22 bc	0.30 ± 0.16 d	0.54 B	0.72 ± 0.08 bc	0.82 ± 0.20 ab	0.55 ± 0.20 bc	0.70 A
**50 mg·L^−1^**	0.84 ± 0.05 a	0.73 ± 0.22 a–c	0.48 ± 0.13 cd	0.68 A	0.84 ± 0.13 ab	0.78 ± 0.19 a–c	0.46 ± 0.17 cd	0.69 A
**100 mg·L^−1^**	0.74 ± 0.11 a–c	0.65 ± 0.18 a–c	0.42 ± 0.29 cd	0.60 AB	1.09 ± 0.26 a	0.46 ± 0.18 cd	0.16 ± 0.06 d	0.57 A
**Mean**	0.80 A	0.63 B	0.40 C		0.88 A	0.69 B	0.39 C	
	**Chlorophyll *b* content (mg·g^−1^ FW)**							
**control object**	0.43 ± 0.07 ab	0.18 ± 0.12 cd	0.10 ± 0.05 d	0.24 C	0.47 ± 0.01 a	0.45 ± 0.10 a	0.21 ± 0.07 bc	0.38 A
**50 mg·L^−1^**	0.50 ± 0.03 a	0.35 ± 0.10 ab	0.17 ± 0.05 d	0.34 A	0.48 ± 0.06 a	0.46 ± 0.11 a	0.20 ± 0.09 bc	0.38 A
**100 mg·L^−1^**	0.42 ± 0.06 ab	0.34 ± 0.11 bc	0.15 ± 0.12 d	0.30 AB	0.55 ± 0.15 a	0.25 ± 0.06 b	0.06 ± 0.07 c	0.29 B
**Mean**	0.45 A	0.29 B	0.14 C		0.50 A	0.39 ± B	0.16 C	
	**Chlorophyll *a/b* ratio**							
**control object**	1.92 ± 0.06 cd	3.45 ± 1.47 a–c	3.64 ± 1.58 ab	3.00 A	1.55 ± 0.16 a	1.83 ± 0.13 a	2.61 ± 0.33 a	2.00 A
**50 mg·L^−1^**	1.67 ± 0.17 d	2.11 ± 0.36 a–d	2.88 ± 0.21 a–d	2.22 B	1.76 ± 0.15 a	1.70 ± 0.07 a	2.57 ± 0.85 a	2.01 A
**100 mg·L^−1^**	1.75 ± 0.14 cd	1.99 ± 0.24 b–d	3.74 ± 1.60 a	2.49 AB	2.00 ± 0.09 a	1.76 ± 0.26 a	15.91 ± 9.57 a	6.56 A
**Mean**	1.78 B	2.52 B	3.42 A		1.77 A	1.76 A	7.03 A	
	**Chlorophylls (*a + b*) content (mg·g^−1^ FW)**							
**control object**	1.26 ± 0.17 a	0.68 ± 0.33 b–e	0.39 ± 0.20 e	0.78 B	1.19 ± 0.09 a-c	1.27 ± 0.29 a	0.76 ± 0.27 bc	1.07 A
**50 mg·L^−1^**	1.34 ± 0.04a	1.07 ± 0.30 ac	0.66 ± 0.19 ce	1.02 A	1.32 ± 0.18 a	1.23 ± 0.29 ab	0.65 ± 0.25 de	1.07 A
**100 mg·L^−1^**	1.16 ± 0.17 ab	0.99 ± 0.29 a–d	0.57 ± 0.39 de	0.91 AB	1.64 ± 0.41 a	0.71 ± 0.24 cd	0.22 ± 0.13 e	0.86 B
**Mean**	1.25 A	0.91 B	0.54 C		1.38 A	1.07 B	0.54 C	
	**Carotenoids content (mg·g^−1^ FW)**							
**control object**	0.23 ± 0.04 a	0.13 ± 0.05 b–d	0.07 ± 0.03 d	0.14 A	0.20 ± 0.01 bc	0.22 ± 0.05 ab	0.12 ± 0.05 c–e	0.18 AB
**50 mg·L^−1^**	0.21 ± 0.02 a	0.17 ± 0.05 a–c	0.11 ± 0.03 b–d	0.16 A	0.23 ± 0.03 ab	0.23 ± 0.04 ab	0.11 ± 0.05 de	0.19 A
**100 mg·L^−1^**	0.19 ± 0.03 ab	0.16 ± 0.04 a–c	0.09 ± 0.06 cd	0.15A	0.28 ± 0.08 a	0.13 ± 0.03 cd	0.05 ± 0.01 e	0.15 B
**Mean**	0.21 A	0.15 B	0.09 C		0.24 A	0.19 B	0.09 C	
	**Chlorophylls/carotenoids ratio**							
**control object**	5.61 ± 0.25 a	5.13 ± 0.90 a	6.20 ± 2.29 a	5.65 A	6.01 ± 0.59 a	5.85 ± 0.34 a	6.24 ± 0.56 a	6.03 A
**50 mg·L^−1^**	6.40 ± 0.32 a	6.49 ± 0.89 a	5.80 ± 0.33 a	6.23 A	5.80 ± 0.24 a	5.26 ± 0.42 a	6.00 ± 1.60 a	5.68 A
**100 mg·L^−1^**	6.22 ± 0.09 a	6.18 ± 0.40 a	6.36 ± 2.43 a	6.25 A	5.88 ± 0.19 a	5.22 ± 0.48 a	4.84 ± 2.99 a	5.31 A
**Mean**	6.08 A	5.93 A	6.12 A		5.90 A	5.44 A	5.69 A	
	**Anthocyanins content (mg·g^−1^ FW)**							
**control object**	0.28 ± 0.02 a	0.18 ± 0.08 a	0.13 ± 0.07 a	0.20 A	0.23 ± 0.06 ab	0.23 ± 0.03 ab	0.14 ± 0.04 c	0.20 A
**50 mg·L^−1^**	0.25 ± 0.04 a	0.14 ± 0.04 a	0.11 ± 0.02 a	0.17 A	0.25 ± 0.04 a	0.16 ± 0.02 bc	0.18 ± 0.06 a–c	0.20 A
**100 mg·L^−1^**	0.25 ± 0.03 a	0.24 ± 0.07 a	0.12 ± 0.02 a	0.20 A	0.25 ± 0.02 a	0.16 ± 0.05 bc	0.16 ± 0.04 bc	0.19 A
**Mean**	0.26 A	0.19 A	0.12 A		0.24 A	0.18 B	0.16 B	
	**Phenolic compounds content (mg·g^−1^ FW)**							
**control object**	9.54 ± 2.24 ab	6.72 ± 1.79 b–d	6.32 ± 2.37 cd	7.53 AB	6.09 ± 2.47 a	9.21 ± 1.03 a	7.04 ± 3.39 a	7.45 A
**50 mg·L^−1^**	7.67 ± 0.14 a–d	6.79 ± 1.63 b–d	5.52 ± 1.12 d	6.66 B	9.66 ± 2.25 a	7.77 ± 3.69 a	9.13 ± 1.91 a	8.85 A
**100 mg·L^−1^**	9.09 ± 1.40 a–c	10.32 ± 1.06 a	6.11 ± 1.50 cd	8.51 A	6.78 ± 1.84 a	8.03 ± 3.27 a	9.88 ± 1.49 a	8.23 A
**Mean**	8.77 A	7.94 A	5.98 B		7.51 A	8.34 A	8.68 A	

Means ± SD in columns and rows for each cultivar tested followed by the same letter do not differ significantly at *p* ≤ 0.05 (Tukey’s test). Upper-case letters refer to the main effects (irrespectively), lower-case letters refer to the interaction between the two studied independent variables.

**Table 2:** Rooting, acclimatization, and flowering of *Chrysanthemum* *× grandiflorum* ‘Lilac Wonder’ and ‘Richmond’ shoots, depending on the AgNPs treatment (0–100 mg·L^−1^).

Concentration of AgNPs	No. of Shoots Transferred to Rooting Medium	No. (%) of Acclimatized and Ex Vitro Cultivated Shoots	No. (%) of Flowering Shoots	No. of Mutants	Frequency of Mutants (%)	No. of Mutations	Frequency of Mutations (%)
			**‘Lilac Wonder’**				
**standard**	25	25 (100 a)	24 (96.0 a)	0	0	0	0
**control object**	100	59 (59 b)	44 (74.6 a)	0	0	0	0
**50 mg·L^−1^**	92	52 (56.5 b)	43 (82.7 a)	1	2.3	1	2.3
**100 mg·L^−1^**	55	55 (100 a)	50 (90.9 a)	5	10	5	10
			**‘Richmond’**				
**standard**	25	25 (100 a)	17 (68.0 a)	0	0	0	0
**control object**	81	81 (100 a)	73 (90.1 a)	1	1.4	1	1.4
**50 mg·L^−1^**	17	17 (100 a)	14 (82.3 a)	0	0	0	0
**100 mg·L^−1^**	1	1 (100 a)	1 (100 a)	0	0	0	0

Data in columns for each cultivar tested followed by the same letter do not differ significantly at *p* ≤ 0.05 (Tukey’s test). Standard—plants propagated by the single-node method on the PGRs-free medium. Control—adventitious shoots regenerated on the AgNPs-free medium.

**Table 3:** Inflorescence characteristics, composition, and content of pigments in ligulate florets of *Chrysanthemum* *× grandiflorum* ‘Lilac Wonder’ and ‘Richmond’, and their mutants, depending on the AgNPs treatment (0–100 mg·L^−1^).

Concentration of AgNPs	Inflorescence Characteristic			Content of Pigments in Ligulate Florets (mg·g^−1^ FW)	
	Color	RHSCC Color Code	Shape	Anthocyanins	Carotenoids
**‘Lilac Wonder’**					
**Standard**	pink	70C/69A *	full, semi-ball	0.65	-
**Individual no. 1** **(50 mg·L^−1^ AgNPs)**	light pink with a burgundy-gold stripe	69B/69D172D/163C	full, semi-ball	0.39	0.40
**Individual no. 2** **(100 mg·L^−1^ AgNPs)**	burgundy-gold	173A/163B	full, semi-ball	0.60	0.34
**Individual no. 3** **(100 mg·L^−1^ AgNPs)**	burgundy-gold	173A/163B	full, semi-ball	0.77	0.46
**Individual no. 4** **(100 mg·L^−1^ AgNPs)**	light burgundy-gold	172D/163C	full, semi-ball	1.26	0.44
**Individual no. 5** **(100 mg·L^−1^ AgNPs)**	light burgundy-gold	172D/163C	full, semi-ball	1.37	0.51
**Individual no. 6** **(100 mg·L^−1^ AgNPs)**	burgundy-gold	173A/163B	full, semi-ball	0.78	0.36
**‘Richmond’**					
**Standard**	purple pink	71B/75D	full, flat	0.84	-
**Individual no. 1** **(0 mg·L^−1^ AgNPs)**	purple pink	71B/75D	full, irregular	1.18	-

Standard—plants propagated by the single-node method on the PGRs-free medium. RHSCC—The Royal Horticultural Society Colour Chart [28]. * inner/outer side of ligulate florets

**Table 4:** Characteristics of molecular products obtained from *Chrysanthemum* *× grandiflorum* ‘Lilac Wonder’ (LW) and ‘Richmond’ (R) analyzed with randomly amplified polymorphic DNA (RAPD) and start codon targeted polymorphism (SCoT) markers.

Cv.	Primer Symbol	Primer Sequence 5′ → 3′	No. of Bands	No. of *Loci*				Total Polymorphic *loci* [%]	No. (%) of Polymorphic Plants	No. of Genotypes
				Total	Mono.	Poly.	Spec.			
				**RAPD**						
**LW** **R**	**R1**	GGG AAT TCG G	588	12	0	12	0	100	23 (27.1)	2
			399	7	2	5	0	71.4	17 (25)	3
**LW**	**R2**	GAC CGC TTG T	425	5	5	0	0	0.0	0 (0.0)	1
**R**			528	9	7	2	0	22.2	18 (26.5)	3
**LW**	**R3**	GCT GCC TCA GG	510	6	6	0	0	0.0	0 (0.0)	1
**R**			414	8	6	2	0	25	3 (4.4)	2
**LW**	**R4**	TAC CCA GGA GCG	509	7	4	2	1	42.9	1 (1.2)	2
**R**			476	10	6	4	0	40.0	15 (22.1)	5
**LW**	**R5**	CAA TCG CCG T	170	2	2	0	0	0.0	0 (0.0)	1
**R**			101	8	0	8	0	100	17 (25)	4
**LW** **R**	**∑**		2202 1918	32 42	17 21	14 21	1 0	- -	24 (28.2) 18 (26.5)	3 13
**LW** **R**	**mean from a single primer**		440.4 383.3	6.4 8.4	3.4 4.2	2.8 4.2	0.2 0.0	28.6 51.7	- -	- -
				**SCoT**						
**LW** **R**	**S1**	CAA CAA TGG CTA CCA CCG	595	7	7	0	0	0.0	0 (0.0)	1
			413	12	3	4	5	75.0	3 (4.4)	3
**LW** **R**	**S2**	CAA CAA TGG CTA CCA CCT	595	7	7	0	0	0.0	0 (0.0)	1
			478	8	7	1	0	12.5	2 (2.9)	2
**LW** **R**	**S3**	CAA CAA TGG CTA CCA CGT	595	7	7	0	0	0.0	0 (0.0)	1
			277	6	4	2	0	33.3	4 (5.9)	3
**LW** **R**	**S4**	ACG ACA TGG CGA CCA ACG	850	10	10	0	0	0.0	0 (0.0)	1
			551	10	7	3	0	30.0	11 (16.2)	4
**LW** **R**	**S5**	ACG ACA TGG CGA CCA TCG	680	12	4	4	4	66.7	1 (1.2)	2
			409	7	6	0	1	14.3	1 (1.5)	2
**LW** **R**	**∑**		3315 2128	43 43	35 27	4 10	4 6	- -	1 (1.2) 17 (25)	2 11
**LW** **R**	**mean from a single primer**		663 425.6	8.6 8.6	7.0 5.4	0.8 2.0	0.8 1.2	13.3 33.0	- -	- -

mono.—monomorphic, poly.—polymorphic, spec.—specific (unique; present in a single band profile).

**Table 5:** Values of Heterozygosity index (H), Polymorphic Information Content (PIC), Effective multiplex ratio (E), Marker Index (MI), Discriminating power (D), and Resolving power (R) of the marker systems used in the study.

Cv.	Primer	H	PIC	E	MI	D	R
	**RAPD**						
LW	R-1	0.49	0.37	6.92	0.003	0.67	6.49
R		0.27	0.40	5.87	0.003	0.30	2.26
LW	R-2	0.0	0.0	0.0	0.0	0.0	0.0
R		0.24	0.41	7.76	0.003	0.26	0.59
LW	R-3	0.0	0.0	0.0	0.0	0.0	0.0
R		0.36	0.38	6.09	0.004	0.42	0.18
LW	R-4	0.20	0.18	7.99	0.002	0.21	0.07
R		0.42	0.35	7.00	0.004	0.51	0.71
LW	R-5	0.0	0.0	0.0	0.0	0.0	0.0
R		0.30	0.40	1.48	0.001	0.97	1.74
**LW**	**mean**	**0.14**	**0.11**	**2.98**	**0.001**	**0.18**	**1.31**
**R**		**0.32**	**0.39**	**5.64**	**0.003**	**0.49**	**1.10**
	**SCoT**						
LW	S-1	0.0	0.0	0.0	0.0	0.0	0.0
R		0.50	0.27	6.07	0.004	0.74	0.32
LW	S-2	0.0	0.0	0.0	0.0	0.0	0.0
R		0.21	0.37	7.03	0.003	0.23	0.06
LW	S-3	0.0	0.0	0.0	0.0	0.0	0.0
R		0.44	0.30	4.07	0.004	0.54	0.15
LW	S-4	0.0	0.0	0.0	0.0	0.0	0.0
R		0.31	0.35	8.10	0.004	0.34	0.74
LW	S-5	0.44	0.35	8.0	0.004	0.56	0.19
R		0.24	0.37	6.02	0.003	0.26	0.03
**LW**	**mean**	**0.09**	**0.07**	**1.6**	**0.001**	**0.11**	**0.04**
**R**		**0.34**	**0.33**	**6.26**	**0.004**	**0.42**	**0.26**

Cv.—cultivar, LW—‘Lilac Wonder’, R—‘Richmond’, RAPD—Randomly Amplified Polymorphic DNA, SCoT—Start Codon Target Polymorphism.

## Data Availability

Data available by e-mail on reasonable request.

## Supplementary Materials

The following supporting information can be downloaded at: www.mdpi.com/article/10.3390/ijms23137406/s1.

## References

[1] Yadollahi A., Arzani K., Khoshghalb H. (2010). The role of nanotechnology in horticultural crops postharvest management. Acta Hortic..

[2] Milewska-Hendel A., Gawecki R., Zubko M., Stróż D., Kurczyńska E. (2016). Diverse influence of nanoparticles on plant growth with a particular emphasis on crop plants. Acta Agrobot..

[3] Fayez K.A., El-Deeb B.A., Mostafa N.Y. (2017). Toxicity of biosynthetic silver nanoparticles on the growth, cell ultrastructure and physiological activities of barley plant. Acta Physiol. Plant..

[4] Singh A., Singh N.B., Afzal S., Singh T., Hussain I. (2018). Zinc oxide nanoparticles: A review of their biological synthesis, antimicrobial activity, uptake, translocation and biotransformation in plants. J. Mater. Sci..

[5] Sanzari I., Leone A., Ambrosone A. (2019). Nanotechnology in plant science: To make a long story short. Front. Bioeng. Biotechnol..

[6] Tymoszuk A., Kulus D. (2020). Silver nanoparticles induce genetic, biochemical, and phenotype variation in chrysanthemum. Plant Cell Tissue Org. Cult..

[7] Tymoszuk A. (2021). Silver nanoparticles effects on in vitro germination, growth, and biochemical activity of tomato, radish, and kale seedlings. Materials.

[8] Yan A., Chen Z. (2019). Impacts of silver nanoparticles on plants: A focus on the phytotoxixity and underlying mechanism. Int. J. Mol. Sci..

[9] Tarafdar J.C., Aishwath O.P., Balra S., Dubey P.N., Mishra B.K. (2015). Nanoparticle production, characterization ant its application to horticultural crops. Compendium of Lectures of Winter School on Utilization of Degraded Land and Soil through Horticultural Crops for Improving Agricultural Productivity and Environmental Quality.

[10] Mandal D., Lalrinchhani (2021). Nanofertilizer and its application in horticulture. J. Appl. Hortic..

[11] Rana R.A., Siddiqui M.N., Skalicky M., Brestic M., Hossain A., Kayesh E., Popov M., Hejnak V., Gupta D.R., Mahmud N.U. (2021). Prospects of nanotechnology in improving the productivity and quality of horticultural crops. Horticulturae.

[12] Tymoszuk A., Miler N. (2019). Silver and gold nanoparticles impact on *in vitro* adventitious organogenesis in chrysanthemum, gerbera and Cape Primrose. Sci. Hortic..

[13] Kulus D., Tymoszuk A. (2021). Gold nanoparticles affect the cryopreservation efficiency of in vitro-derived shoot tips of bleeding heart. Plant Cell Tissue Org. Cult..

[14] Tymoszuk A., Wojnarowicz J. (2020). Zinc oxide and zinc oxide nanoparticles impact on *in vitro* germination and seedling growth in *Allium cepa* L.. Materials.

[15] Patlolla A.K., Berry A., May L., Tchounwou P.B. (2012). Genotoxicity of silver nanoparticles in *Vicia faba*: A pilot study on the environmental monitoring of nanoparticles. Int. J. Environ. Res. Public Health..

[16] Abdelsalam N.R., Abdel-Megeed A., Ali H.M., Salem M.Z.M., Al-Hayali M.F.A., Elshikh M.S. (2018). Genotoxicity effects of silver nanoparticles on wheat (*Triticum aestivum* L.) root tip cells. Ecotoxicol. Environ. Saf..

[17] Speranza A., Crinelli R., Scoccianti V., Taddei A.R., Iacobucci M., Bhattacharya P., Ke P.C. (2013). In vitro toxicity of silver nanoparticles to kiwifruit pollen exhibits peculiar traits beyond the cause of silver ion release. Environ. Pollut..

[18] Tripathi D.K., Shweta, Singh S., Singh S., Pandey R., Singh V.P., Sharma N.C., Prasad S.M., Dubey N.K., Chauhan D.K. (2016). An overview on manufactured nanoparticles in plants: Uptake, translocation, accumulation and phytotoxicity. Plant Physiol. Biochem..

[19] Čapek J., Roušar T. (2021). Detection of oxidative stress induced by nanomaterials in cells–the roles of reactive oxygen species and glutathione. Molecules.

[20] Horie M., Tabei Y. (2021). Role of oxidative stress in nanoparticles toxicity. Free Radic. Res..

[21] Su J., Jiang J., Zhang F., Liu Y., Ding L., Chen S., Chen F. (2019). Current achivements and future prospects in the genetic breeding of chrysanthemum: A review. Hortic. Res..

[22] Miler N., Jedrzejczyk I., Jakubowski S., Winiecki J. (2021). Ovaries of chrysanthemum irradiated with high-energy photons and high-energy electrons can regenerate plants with novel traits. Agronomy.

[23] Zalewska M., Tymoszuk A., Miler N. (2011). New chrysanthemum culivars as a result of *in vitro* mutagenesis with the application of different explants types. Acta Sci. Pol. Hortorum Cultus.

[24] Holme I.B., Gregersen P.L., Brinch-Pedersen H. (2019). Induced genetic variation in crop plants by random or targeted mutagenesis: Convergence and differences. Front. Plant Sci..

[25] Mir A.S., Maria M., Muhammad S., Ali S.M., Maia R.T., de Araujo Campos M. (2020). Potential of mutation breeding to sustain food security. Genetic Variation.

[26] Zalewska M., Miler N., Tymoszuk A., Drzewiecka B., Winiecki J. (2010). Results of mutation breeding activity on *Chrysanthemum* × *grandiflorum* (Ramat.) Kitam. in Poland. EJPAU.

[27] Shin J., Bae S., Seo P.J. (2020). De novo shoot organogenesis during plant regeneration. J. Exp. Bot..

[28] RHSCC (1966). The Royal Horticultural Society Colour Chart.

[29] Miler N., Kulus D. (2018). Microwave treatment can induce chrysanthemum phenotypic and genetic changes. Sci. Hortic..

[30] Barbasz A., Kreczmer B., Oćwieja M. (2016). Effects of exposure of callus cells of two wheat varieties to silver nanoparticles and silver salt (AgNO_3_). Acta Physiol. Plant..

[31] Cvjetko P., Zovko M., Peharec Štefanić P., Biba R., Tkalec M., Domijan A.M., Vinković Vrček I., Letofsky-Papst I., Šikić S., Balen B. (2018). Phytotoxic effects of silver nanoparticles in tobacco plants. Environ. Sci. Pollut. Res..

[32] Chahardoli A., Karimi N., Ma X., Qalekhani F. (2020). Effects of engineered aluminium and nickel oxide nanoparticles on the growth and antioxidant defense systems of *Nigella arvensis* L.. Sci. Rep..

[33] Zalewska M. (1995). Somatic Mutagenesis in Chrysanthemum (Dendranthema Grandiflora Tzvelev) Induced In Vivo and In Vitro by X and Gamma Radiation (In Polish).

[34] Broertjes C., Roest S., Bokelmann G.S. (1976). Mutation breeding of *Chrysanthemum morifolium* Ram. using *in vivo* and *in vitro* adventitious bud techniques. Euphytica.

[35] Broertjes C., Keen A. (1980). Adventitious shoots: Do they develop from one cell?. Euphytica.

[36] Nabeshima T., Yang S.-J., Ohno S., Honda K., Deguchi A., Doi M., Tatsuzawa F., Hosokawa M. (2017). Histogen layers contribiuting to adventitious bud formation are determined by their cell division activities. Front. Plant Sci..

[37] Lema-Rumińska J., Zalewska M. (2004). Studies on flower pigment of chrysanthemum mutants: Nero and Wonder Groups. Acta Sci. Pol. Hortorum Cultus.

[38] Lema-Rumińska J., Zalewska M. (2005). Changes in flower colour among Lady Group of *Chrysanthemum* × *grandiflorum* (Ramat.) Kitam. as a result of mutation breeding. Fol. Hortic. Ann..

[39] Lema-Rumińska J., Kulus D., Tymoszuk A., Varejão J.M.T.B., Bahcevandziev K. (2019). Profile of secondary metabolites and genetic stability analysis in new lines of *Echinacea purpurea* (L.) Moench micropropagated via somatic embryogenesis. Industr. Crops Prod..

[40] Kulus D., Rewers M., Serocka M., Mikuła A. (2019). Cryopreservation by encapsulation-dehydration affects the vegetative growth of chrysanthemum but does not disturb its chimeric structure. Plant Cell Tissue Org. Cult..

[41] Shukla R.K., Badiye A., Vajpayee K., Kapoor N. (2021). Genotoxic potential of nanoparticles: Structural and functional modifications in DNA. Front. Genet..

[42] Kulus D., Tymoszuk A., Jędrzejczyk I., Winiecki J. (2022). Gold nanoparticles and electromagnetic irradiation in tissue culture systems of bleeding heart: Biochemical, physiological, and (cyto)genetic effects. Plant Cell Tissue Organ Cult..

[43] Schum A. (2003). Mutation breeding in ornamentals: An efficient breeding method?. Acta Hortic..

[44] Jerzy M., Zalewska M. (1997). Flower colour recurrence in chrysanthemum and gerbera mutants propagated *in vitro* with meristems and leaf explants. Acta Hortic..

[45] Rogozin I.B., Pavlov Y.I. (2003). Theoretical analysis of mutation hotspots and their DNA sequence context specificity. Mutat. Res..

[46] Silva G.S., Souza M.M. (2013). Genomic *in situ* hybridization in plants. Genet. Molec. Res..

[47] Murashige T., Skoog F. (1962). A revised medium for rapid growth and bioassays with tobacco tissue cultures. Physiol. Plant..

[48] Domeradzka-Gajda K., Nocuń M., Roszak J., Janasik B., Quarles C.D., Wąsowicz W., Grobelny J., Tomaszewska E., Celichowski G., Ranoszek-Soliwoda K. (2017). A study on the *in vitro* percutaneous absorption of silver nanoparticles in combination with aluminum chloride, methyl paraben or di-n-butyl phthalate. Toxicol. Lett..

[49] Pudlarz A., Czechowska E., Ranoszek-Soliwoda K., Tomaszewska E., Celichowski G., Grobelny J., Szemraj J. (2018). Immobilization of recombinant human catalase on gold and silver nanoparticles. Appl. Biochem. Biotechnol..

[50] Lichtenthaler H.K. (1987). Chlorophylls and carotenoids: Pigments of photosynthetic biomembranes. Method. Enzymol..

[51] Harborne J.B. (1967). Comparative Biochemistry of the Flavonoids. Phytochemistry.

[52] Waterhouse A.L., Wrolstad R.E. (2001). Determination of total phenolics. Current Protocols in Food Analytical Chemistry.

[53] Homaee M.B., Ehsanpour A.A. (2016). Silver nanoparticles and silver ions: Oxidative stress responses and toxicity in potato (*Solanum tuberosum* L.) grown in vitro. Hortic. Environ. Biotechnol..

[54] Bradford M.M. (1976). A rapid and sensitive method for the quantitation of microgram quantities of protein utilizing the principle of protein-dye binding. Anal. Biochem..

[55] Giannopolitis C.N., Ries S.K. (1977). Superoxide dismutases I. Occurrence in higher plants. Plant Physiol..

[56] Maehly A.C., Chance B., Glick D. (1954). The assay of catalases and peroxidases. Methods of Biochemical Analysis.

[57] Nowogórska A., Patykowski J. (2015). Selected reactive oxygen species and antioxidany enzymes in common bean after *Pseudomonas syringae* pv. phaseolicola and Botrytis cinerea infection. Acta Physiol. Plant..

[58] Williams J.G.K., Kubelik A.R., Livak K.J., Rafalski J.A., Tingey S.V. (1990). DNA polymorphisms amplified by arbitrary primers are useful as genetic markers. Nucl. Acids Res..

[59] Collard B.C.Y., Mackill D.J. (2009). Start codon targeted (SCoT) polymorphism: A simple, novel DNA marker technique for generating gene-targeted markers in plants. Plant Mol. Biol. Rep..

[60] Amiryousefi A., Hyvönen J., Poczai P. (2018). iMEC: Online Marker Efficiency Calculator. Appl. Plant Sci..

[61] Nei M., Li W.S. (1979). Mathematical model for studying genetic variation in terms of restriction nucleases. Proc. Natl. Acad. Sci. USA.

[62] Peakall R., Smouse P.E. (2012). GenAlEx 6.5: Genetic analysis in Excel. Population genetic software for teaching and research-an update. Bioinformatics.

