# Supplementary material for: Effect of Silver Nanoparticles on the In Vitro Regeneration, Biochemical, Genetic, and Phenotype Variation in Adventitious Shoots Produced from Leaf Explants in Chrysanthemum
Source: Int J Mol Sci. 2022 Jul 3;23(13):7406. doi: 10.3390/ijms23137406 (PMC9266331; doi:10.3390/ijms23137406)
Supplement: Supplementary file 1 [file ijms-23-07406-s001.zip › ijms-1777101-supplementary.pdf]

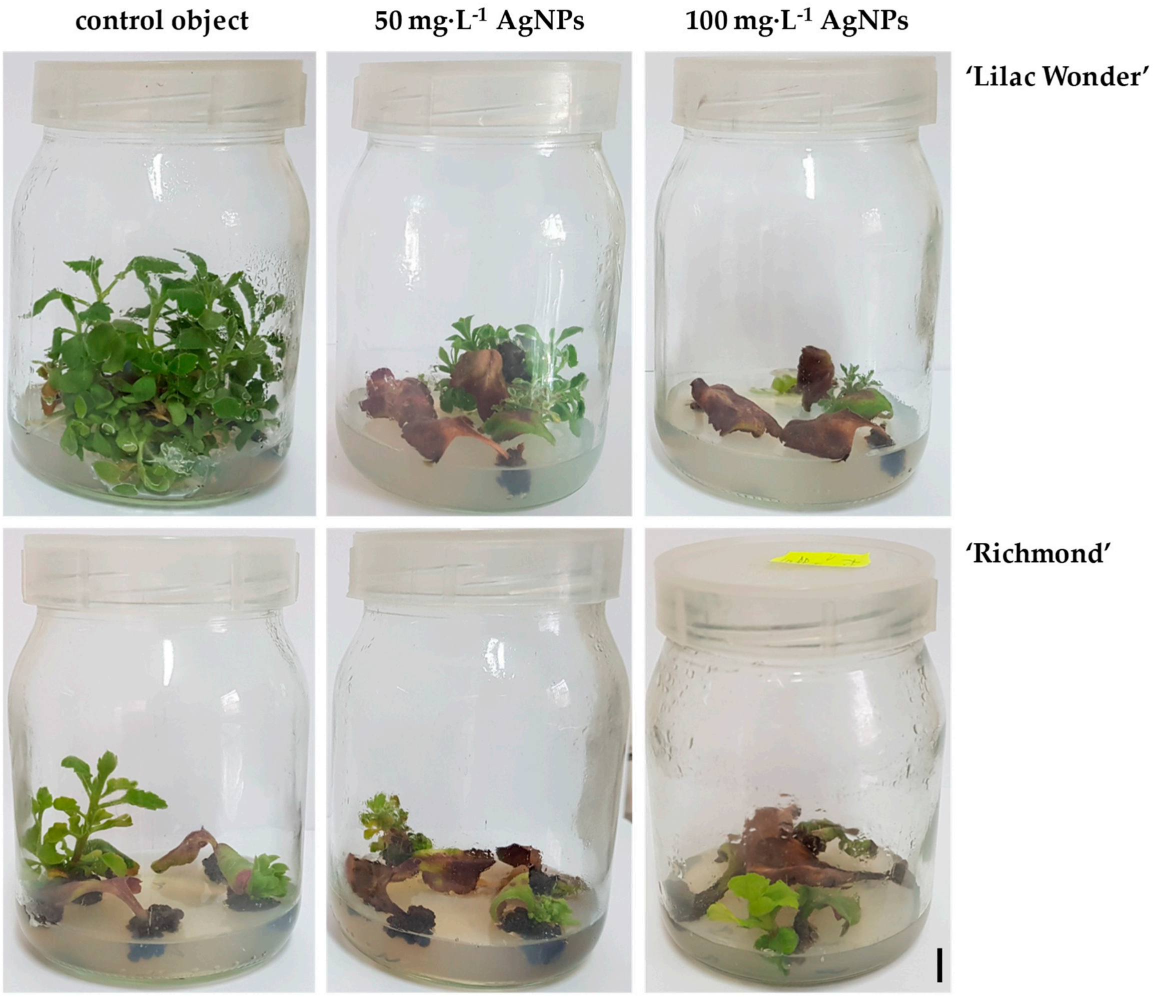

Figure S1. Adventitious shoots regeneration on the inoculated *Chrysanthemum* × *grandiflorum* 'Lilac Wonder' and 'Richmond' leaf explants cultured on the modified MS medium with 0.6 mg·L^{-1} BAP and 2 mg·L^{-1} IAA in the ninth week of culture, depending on the AgNPs treatment (0–100 mg·L^{-1}); bar = 1 cm.

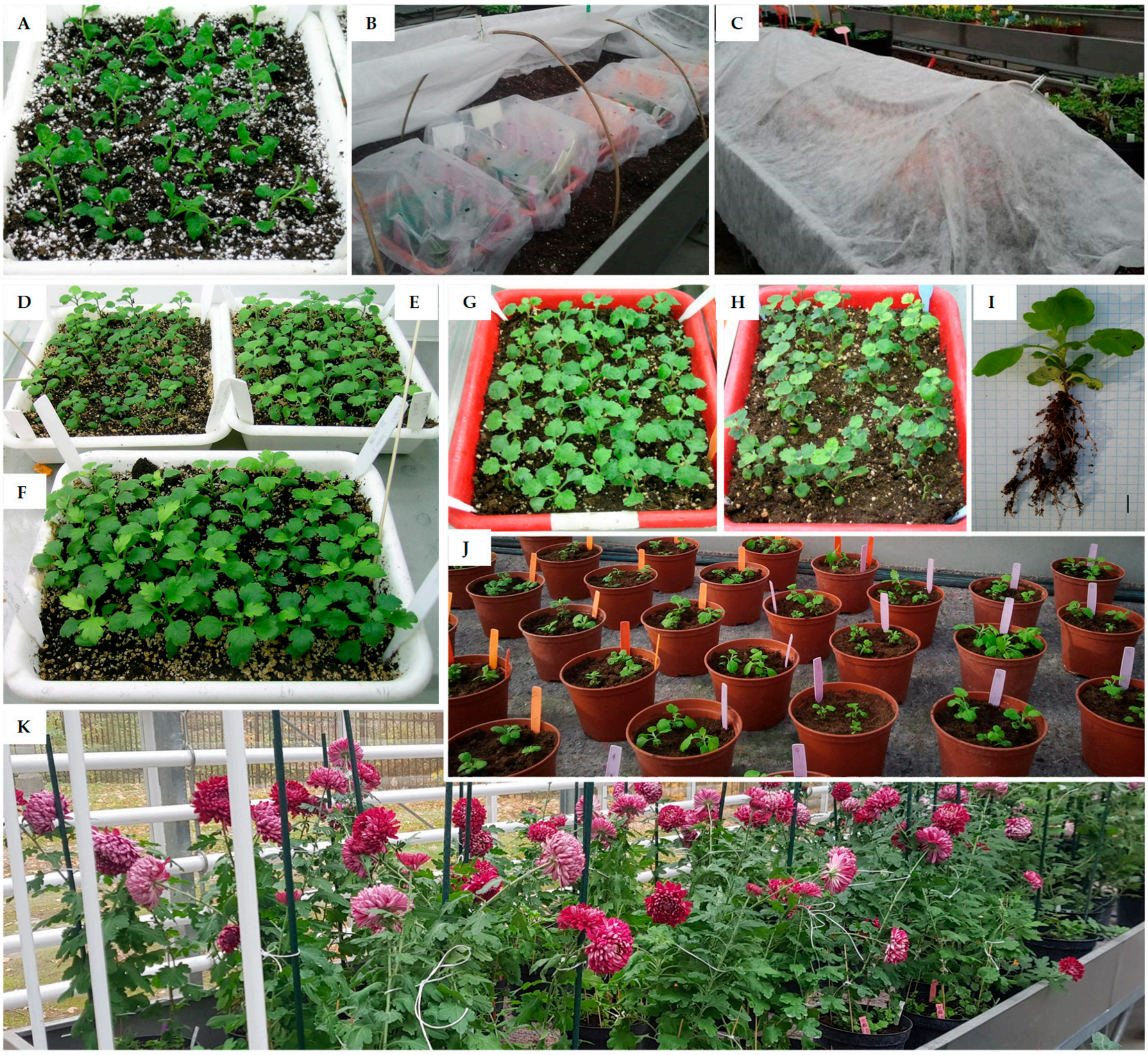

Figure S2. Acclimatization and *ex vitro* growth in greenhouse of *Chrysanthemum* × *grandiflorum* 'Lilac Wonder' and 'Richmond' tested plants: *in vitro*-rooted microshoots planted to plastic trays filled with a mixture of peat substrate and perlite (A); microshoots covered with perforated transparent foil (B) and geo-cover (C) during acclimatization; acclimatized 'Lilac Wonder' plants depending on the AgNPs treatment: control (D), 50 mg·L^{-1} AgNPs (E), and 100 mg·L^{-1} AgNPs (F); acclimatized control 'Richmond' plants (G); acclimatized 50 mg·L^{-1}AgNPs-treated 'Richmond' plants (H); acclimatized 100 mg·L^{-1} AgNPs-treated 'Richmond' plant (I); plants transferred after acclimatization to plastic pots for further cultivation (J); plants at the full floweing stage (K); bar = 1 cm.

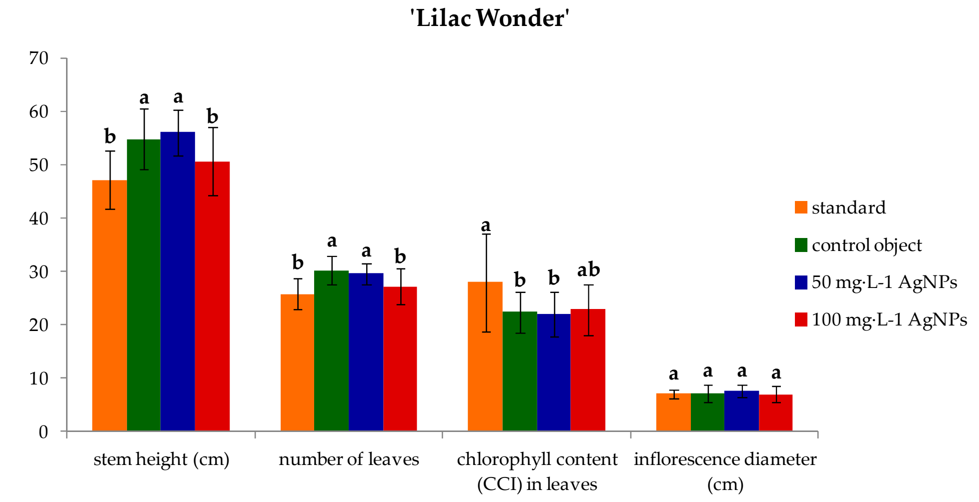

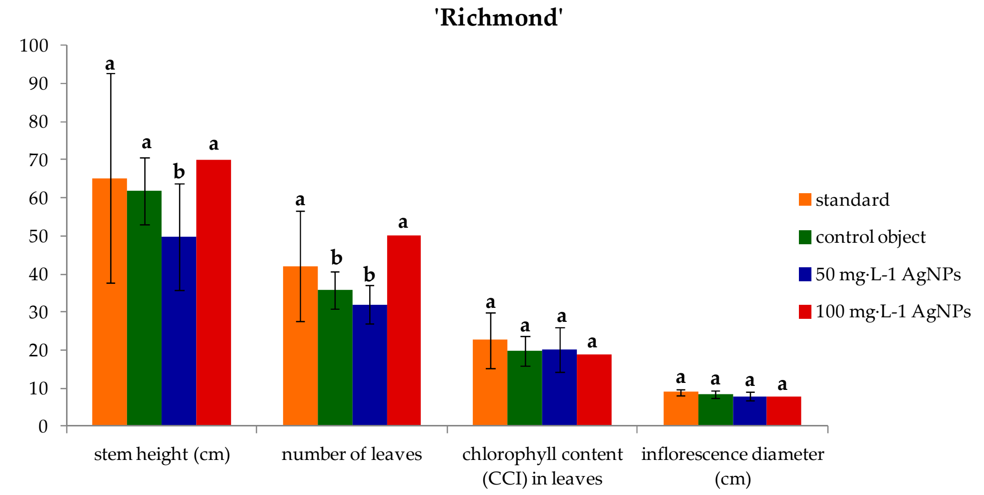

Figure S3. Biometric parameters and chlorophyll content of standard plants and *ex vitro*-cultivated adventitious shoots of *Chrysanthemum* × *grandiflorum* 'Lilac Wonder' and 'Richmond' regenerated from leaf explants cultured *in vitro* on the modified MS medium with 0.6 mg·L^{-1} BAP and 2 mg·L^{-1} IAA, depending on the AgNPs treatment (0–100 mg·L^{-1}). Means ± SD on graphs for each cultivar tested followed by the same letter do not differ significantly at $p \leq 0.05$ (Tukey's test). Standard – plants propagated by the single node method on the PGRs-free medium. Control object – adventitious shoots regenerated on the AgNPs-free medium.

Table S1. Analysis of molecular variance (AMOVA) in the two studied chrysanthemum cultivars.

Source of variation	'Lilac Wonder'		'Richmond'	
	RAPD	SCoT	RAPD	SCoT
Among Populations [%]	1	0	32	10
Within populations [%]	99	100	68	90
Φ_{PT}	0.012	-0.029	0.325	0.104
